# Multiomic analysis for optimization of combined focal and immunotherapy protocols in murine pancreatic cancer

**DOI:** 10.7150/thno.73218

**Published:** 2022-11-14

**Authors:** James Wang, Brett Z. Fite, Aris J. Kare, Bo Wu, Marina Raie, Spencer K. Tumbale, Nisi Zhang, Ryan R. Davis, Clifford G. Tepper, Sharon Aviran, Aaron M. Newman, Daniel A. King, Katherine W. Ferrara

**Affiliations:** 1 Department of Radiology, Stanford University, Palo Alto, CA 94305, USA; 2 Department of Bioengineering, Stanford University, Palo Alto, CA 94305, USA; 3 Department of Pathology and Laboratory Medicine, University of California Davis, School of Medicine, Sacramento, CA 95817, USA; 4 Department of Biochemistry and Molecular Medicine, University of California Davis, School of Medicine, Sacramento, CA 95817, USA; 5 Department of Biomedical Engineering, University of California Davis, Davis, CA 95616, USA; 6 Institute for Stem Cell Biology and Regenerative Medicine, Stanford University, Palo Alto, CA, 94305, USA; 7 Department of Biomedical Data Science, Stanford University, Palo Alto, CA 94305, USA; 8 Division of Medical Oncology/Hematology, Northwell Health Cancer Institute, New Hyde Park, NY 10042 USA

**Keywords:** Combination immunotherapy, Focused ultrasound, Digital cytometry, Spectral cytometry, Sequencing

## Abstract

**Background:** Although combination immunotherapies incorporating local and systemic components have shown promising results in treating solid tumors, varied tumor microenvironments (TMEs) can impact immunotherapeutic efficacy.

**Method:** We designed and evaluated treatment strategies for breast and pancreatic cancer combining magnetic resonance-guided focused ultrasound (MRgFUS) ablation and antibody therapies. With a combination of single-cell sequencing, spectral flow cytometry, and histological analyses, we profiled an immune-suppressed KPC (Kras^+/LSL-G12D^; Trp53^+/LSL-R172H^; Pdx1-Cre) pancreatic adenocarcinoma (MT4) model and a dense epithelial neu deletion (NDL) HER2^+^ mammary adenocarcinoma model with a greater fraction of lymphocytes, natural killer cells and activated dendritic cells. We then performed gene ontology analysis, spectral and digital cytometry to assess the immune response to combination immunotherapies and correlation with survival studies.

**Result:** Based on gene ontology analysis, adding ablation to immunotherapy enriched immune cell migration pathways in the pancreatic cancer model and extensively enriched wound healing pathways in the breast cancer model. With CIBERSORTx digital cytometry, aCD40 + aPD-1 immunotherapy combinations enhanced dendritic cell activation in both models. In the MT4 TME, adding the combination of aCD40 antibody and checkpoint inhibitors (aPD-1 and aCTLA-4) with ablation was synergistic, increasing activated natural killer cells and T cells in distant tumors. Furthermore, ablation with immunotherapy upregulated critical Ly6c myeloid remodeling phenotypes that enhance T-cell effector function and increased granzyme and protease encoding genes by as much as 100-fold. Ablation combined with immunotherapy then extended survival in the MT4 model to a greater extent than immunotherapy alone.

**Conclusion:** In summary, TME profiling informed a successful multicomponent treatment protocol incorporating ablation and facilitated differentiation of TMEs in which ablation is most effective.

## Introduction

Immunotherapies, including checkpoint inhibitors and immune agonists, have reshaped the modern landscape of cancer treatment 1. Reactivating the immune system to eradicate cancer involves modulating multiple immune pathways, motivating the rationale for combinatorial immunotherapy 2. Nevertheless, combination strategies have not been broadly effective in solid tumors 3. Combining immune checkpoint inhibitors (ICIs) aPD-1 with aCTLA-4 has improved survival in metastatic melanoma when compared to monotherapy, yet non-responding patients and adverse events persist, and only a small fraction of patients with breast and pancreatic cancer exhibit a complete response 3, 4. Genomic and immunohistochemical analyses of patient tumor tissues treated with ICIs have revealed that tumor microenvironment (TME) subtypes are associated with treatment outcomes 5, 6.

Here, we combine multiple techniques (single-cell RNA sequencing, bulk RNA sequencing with CIBERSORTx, and spectral cytometry), each with their own unique set of advantages, to assess naïve tumor phenotype, cell type population dynamics and cell-specific treated phenotypes, respectively. Single-cell RNA sequencing (scRNA-seq) assays mRNA (thousands of parameters per cell) for thousands of cells and is used to characterize immune cell composition and gene expression within the TME. We assess checkpoint genes and aCD40 expression using scRNA-seq and directly compare UMAPs generated by scRNA-seq and spectral cytometry in the untreated cohort. Bulk RNA sequencing is analyzed with CIBERSORTx, a cost-effective approach for “digital cytometry” that has been shown to enumerate cell composition from bulk tissue gene expression profiles (GEPs) 7-9. However, CIBERSORTx deconvolution relies on the assumption that tumor infiltrating immune cells share similar genetic signatures to other tissue-resident immune cells and the deconvolution matrices for mice are limited. We use the LM22M matrix, a modified version of the LM22 matrix 10 originally derived from human assays to assess the impact of treatments. Spectral cytometry measures the full emission profile of fluorochromes across 64 detectors in the 360-830 nm wavelength range 11 and can quantify protein expression levels for millions of cells in a single sample. We add spectral cytometry to study cell surface markers that were not specifically addressed in the creation of the LM22 matrix to measure expression profiles on distinct immune subsets as a function of treatment. By using these techniques in parallel, we avoid the disadvantages of any single approach and are able to gain a more complete understanding of leukocyte response to immunotherapy. We also demonstrate the feasibility of first profiling treatment naïve TME on the transcriptomic level as a reference from which to structure cancer treatment planning, with subsequent treatment combinations assayed with bulk sequencing and spectral cytometry due to the lower cost and complexity.

Pancreatic ductal adenocarcinoma (PDAC) is the third leading cause of cancer mortality, with only 9% of patients presenting with tumor localized to the primary site. As a result, the vast majority of patients with pancreatic cancer have unresectable tumors at the time of diagnosis. PDAC is classically resistant to immunotherapy and our particular goal is to optimize treatment in this disease. CD40 is expressed on a subset of pancreatic cancer cells and the overwhelming majority of peritumoral lymphocytes 12, 13. For PDAC, the agonist CD40 (aCD40) monoclonal antibody promotes stromal degradation, dendritic cell (DC) maturation and alters macrophage phenotype, therefore providing an attractive approach for immunotherapy 14, 15. aCD40 has received orphan drug status for the treatment of pancreatic cancer adding to the significance of the evaluation of combinations. Recent studies combining multiple components (e.g. chemotherapy or radiation + aCD40 + aPD-1) have achieved significant, but not curative, responses 16, 17. Combining gemcitabine and Abraxane with aCD40 and aPD-1 immunotherapy yielded promising results, particularly in Phase 1b clinical trials 18. In Phase 2, the primary endpoint of one-year overall survival greater than 35% was met when combining gemcitabine and Abraxane with either aPD-1 or aCD40 therapy, but not with the combination 18. Thus, there is an opportunity to enhance the efficacy of aCD40 protocols in PDAC. A subpopulation of myeloid cells that are Ly6c2^hi^, Ccl7^hi^, Mrc1^hi^ has been implicated in response of pancreatic cancer to aCD40 19, and therefore we particularly examine this cellular subset.

Impaired PDAC response to immunotherapy can be attributed in part to a unique TME with dense fibrosis and low immune cell density when compared to other solid tumors 20, 21. Because of the variability of the TME across cancer types, we explore the feasibility of personalizing treatment planning based on treatment-naïve TME characterization and apply a multi-omics approach in order to understand the treatment impact on immune populations. We focus on protocols that incorporate thermal ablation as thermal ablation is approved for and has impacted multiple clinical applications in cancer therapy and neurological disorders, and ablation of poorly perfused fibrotic tumors is a rational approach 22. Magnetic resonance guided focused ultrasound (MRgFUS) ablation is a non-pharmacological therapeutic method to debulk tumor, induce immunogenic cell death (ICD), and stimulate damage-associated molecular pattern (DAMP) production. This technique has a wide safety margin and the ability to precisely control spatial and thermal dose and is under evaluation in breast and pancreatic cancer (NCT04298242, NCT05291507, NCT02407613), where the intent of current protocols is to establish safety prior to the initiation of combined ablation-immunotherapy trials 23. MRgFUS ablation in pancreatic cancer was shown to reduce the visual analog pain score from 7 to 3 across 6 patients without adverse effects and demonstrated negligible tumor regrowth in the ablated region on 6-month follow-up 24. In broader clinical trials, MRgFUS ablation has also treated intermediate-risk prostate tumors in more than 90% of the patients without major adverse events 25. In pre-clinical studies, focused ultrasound has augmented immunotherapy and improved survival benefits by leveraging ablation-induced immune responses 26-29. In the context of TME immune cell dynamics, MRgFUS ablation induces DC stimulation 30 and macrophage infiltration 31, which can also be modulated with aCD40 17. Herein, we hypothesize that MRgFUS tumor debulking and TME disruption provide an opportunity to synergize with aCD40 immunotherapy combinations specific to TME subtype and induce non-redundant immune cell activation.

TME subtypes are known to impact treatment efficacy and prognosis 32. In order to evaluate the effect of the TME and treatment components, we contrast response across two tumor models, including a pancreatic and breast tumor model. The models are distinct in leukocyte composition and the density of tumor cells and stromal components. The multi-site implanted KPC pancreatic cancer mouse model (Kras^+/LSL-G12D^; Trp53^+/LSL-R172H^; Pdx1-Cre model) contains a dense stroma and immune-suppressed environment similar to human PDAC 16. In fact, human PDAC has a tumor cellularity ranging from 5-20% 20, 21. We contrast this model with that of the murine neu deletion (NDL) HER2^+^ mammary adenocarcinoma model, which is composed of a dense tumor network (tumor cellularity >80%) with a greater fraction of lymphocytes, natural killer (NK) cells and plasmacytoid dendritic cells (pDCs) surrounded by fat cells 33. We evaluate whether the profound differences in the TME between breast and pancreatic cancer will result in a distinct response to ablation and immunotherapy combinations.

**Figure 1:**
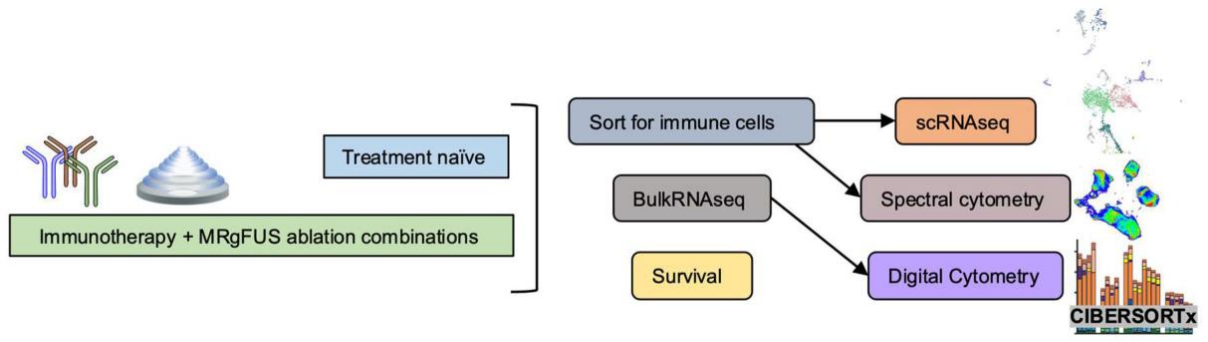
** Schematic of immune effects generated by the combination of ablation and immunotherapy.** Overview of the study. Single-cell RNA sequencing (scRNAseq) and spectral cytometry profiled treatment-naïve tumors to guide combination treatment strategies while next generation sequencing based analyses, such as gene ontology analysis and digital cytometry of treatment combinations, were compared with spectral cytometry characterizations. Tumors were sorted for immune cells prior to scRNAseq and spectral cytometry.

We first profile the tumor immune microenvironment with hematoxylin and eosin (H&E) staining and scRNAseq to characterize the naïve TME immune cell landscape. Based on the naïve TME immune cell profile, we then design treatment strategies to integrate with an MRgFUS ablation protocol (Figure 1). We first evaluate combinations of one or two treatment components (spanning ablation, aCD40 and checkpoint inhibition) in both tumor models, and based on the results, focus on the combination of three to four treatment components in the more aggressive pancreatic cancer model. We characterize combination treatment efficacy via high-throughput bulk RNA sequencing and spectral cytometry to evaluate whether ablation combined with immunotherapies, including aCD40, aPD-1 and aCTLA-4 (with the combination of all three immunotherapies denoted as CP4), is effective. Finally, we evaluate efficacy of the treatment strategies we designed based on our initial TME immune cell profiling.

## Results

### Single-cell RNA sequencing of naïve tumors highlights differences in the TME

**Figure 2:**
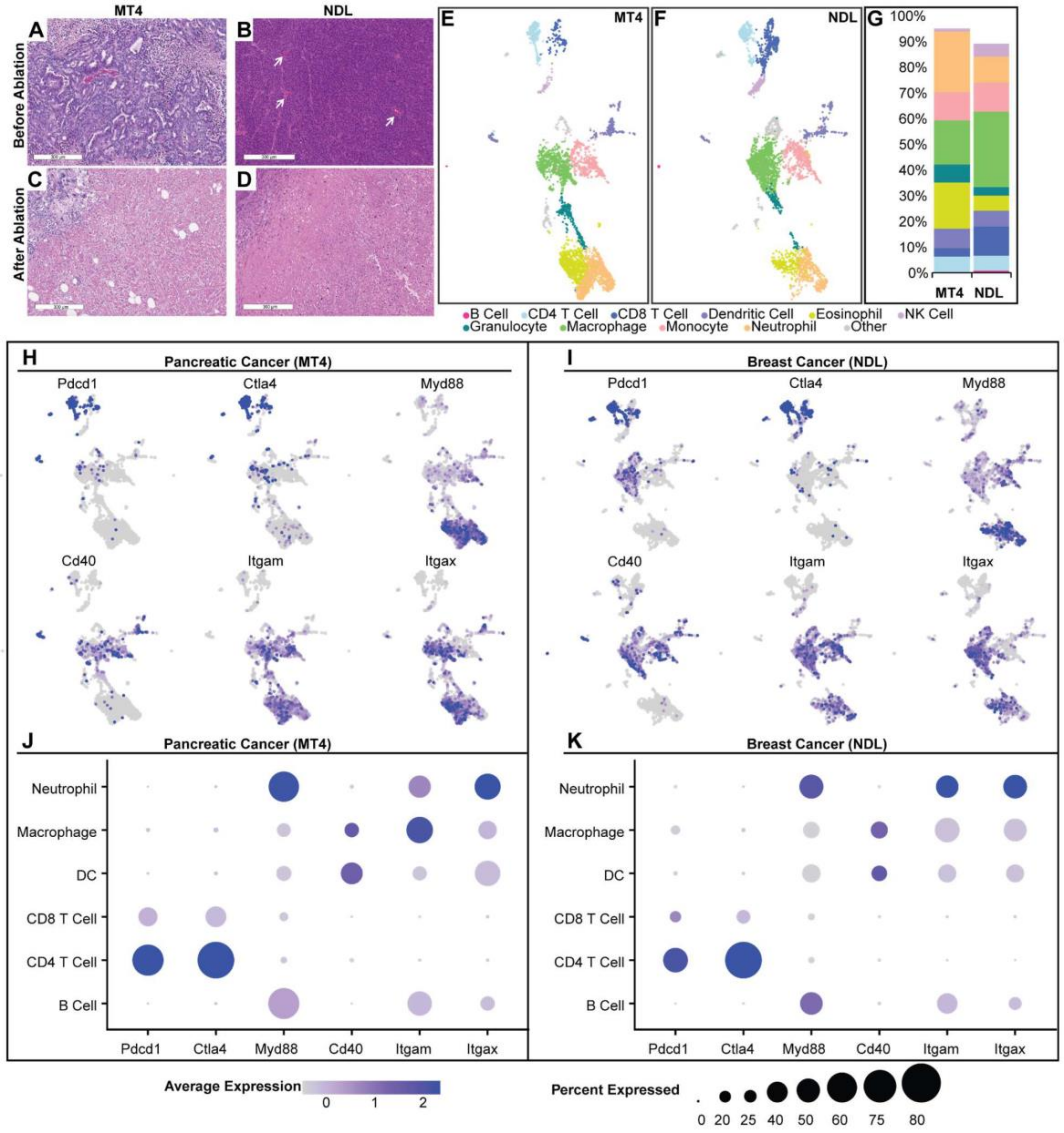
** Analysis of the naïve tumor microenvironment of MT4 pancreatic and NDL breast cancer models reveals distinct TMEs and immunological signatures**. A-D) Hematoxylin and eosin staining before (A-B) and after (C-D) ablation in MT4 (A, C) and NDL (B, D) models. A) MT4 pancreatic ductal adenocarcinoma has decreased cellularity compared to B) NDL mammary adenocarcinoma, which is comparatively well vascularized (white arrows) with scattered leucocytes (black dots). E-K) Results of single-cell RNA sequencing, including Uniform Manifold Approximation and Projection (UMAP) plots for the MT4 (E) and NDL (F) tumors. B cells (dark pink) were CD19^+^, CD79^+^ and Ly6d^+^. T cells were CD3e^+^, with CD8^+^ (dark blue) and CD4^+^ (light blue) T cell subsets defined by CD8a and CD4, respectively. NK cells (lavender) were Klrb1b^+^ and Klrb1c^+^ (NK1.1^+^). Eosinophils (yellow) were Siglec-F^+^. Neutrophils (light orange) were Ly6g^+^. Monocytes (light pink) were Ly6c^+^, Ccr2^+^, Mrc1^+^, and Ccl9^+^. Macrophages (green) were Itgam^+^ and Adgre1^+^. Dendritic cells (purple) were Itgax^+^, H2-Ab1^+^, Fcgr1^+^, Ly6g^-^, Siglecf^-^, Klrb1c^-^. Granulocytes (turquoise) were Tmem189^+^, Sap30^+^, and Idha^+^. G) Quantitative summary of immune cells within each tumor model. The MT4 model has a smaller faction of CD8^+^ T cells, macrophages and NK cells compared to the NDL model. H-K) Gene expression distribution and levels across UMAP cell clusters in the MT4 (H, J) and NDL (I, K) models. In the MT4 model, overall cellular Myd88 expression levels are higher, and CD40 expression is higher in dendritic cell (DC) clusters compared to the NDL model. The MT4 model also has a greater fraction of both CD4^+^ and CD8^+^ T cells expressing PD-1, CD8^+^ T cells expressing CTLA4, and DCs expressing CD40. Scale bars represent 300 µm.

Tumor ablation inherently disrupts the TME and creates a localized immune response that is, in part, a result of wound healing 27. However, the numbers and phenotypes of resident immune cells in the TME can alter immune response following ablation. We first explored the two TME subtypes: the MT4 PDAC model, known to be immunosuppressed 16, and the NDL breast tumor model, which is mildly inflamed 33. MT4 pancreatic tumors have a dense stroma and sparse tumor cells based on H&E (Figure 2A). In contrast, in a fully differentiated HER2^+^ breast adenocarcinoma model, the tumor cell density is high with minimal intercellular stroma (Figure 2B). Focused ultrasound ablation eliminated tumor cells within the ablation zone in each phenotype (MT4 (Figure 2C) and NDL (Figure 2D)) and promoted immune cell infiltration within the ablated region and boundary layer.

To characterize the phenotype of infiltrating immune cells, we resected the tumors and used fluorescently activated cell sorting (FACS) to isolate live, CD45^+^ immune cells for scRNAseq on the 10x sequencing platform. Using the Seurat pipeline 34, we performed hyperparameter tuning by iterating to find the combination of k-nearest number, resolution, and prune parameters that minimized within-cluster-variance. We then used differentially expressed genes across clusters with canonical cell markers to annotate each cluster.

Compared with the NDL model, we found that the MT4 pancreatic model contained higher proportions of DCs, eosinophils and neutrophils, with lower proportions of macrophages, CD8^+^ T and NK cells (Figure 2E-G). This result is consistent with the literature 35, 36. We interrogated the gene expression of each annotated cell cluster. Expression of PD-1 (Pdcd1) and CTLA-4 encoding genes was higher in MT4 (Figure 2H) as compared with NDL (Figure 2I) tumors. Broken down by cell type, the MT4 model had a greater fraction of both CD4^+^ and CD8^+^ T cells expressing PD-1 (Figure 2J) compared to the NDL model (Figure 2K), although the expression level per cell was greater on CD8^+^ T cells in the NDL model. Therefore, we incorporated both checkpoint inhibitors in studies involving MT4 tumors. In addition to the expression of checkpoint-encoding genes, in both models, we found that granulocytes and myeloid cells expressed Myd88 pathway genes, which is central in the toll-like receptor (TLR) activation pathway (Figure 2J-K). CD40 was primarily expressed in DCs in the pancreatic model and macrophages in the breast cancer model (Figure 2J-K).

The myeloid compartment in treatment-naïve tumors clustered into distinct sub-populations including macrophages, monocytes, and DC subtypes (Figure 2 and Figure S1). Within the myeloid compartment, monocytes expressed a high level of Ly6c2, Ccl7, and Mrc1 (Figure S1), genes which have been implicated in the response of pancreatic cancer to aCD40 19. Of the top 40 genes within the monocyte cluster, compared with other clusters, Ly6c2 had an average log_2_ fold change of 2.16 in the MT4 pancreatic cancer model and 1.17 in the NDL breast cancer model. When compared to other clusters, the monocyte cluster (Figure S1) also differentially expressed genes such as Itgam (Cd11b), Fcgr1, Csf1r (Cd115), and Ccr2, which were key defining markers. Also expressed in this cluster were Ccl6 and Ccl9 (Figure S2). As a result of this analysis, checkpoint inhibition and aCD40 immunotherapies were selected for combination with ablation in our studies, and we selected monocytes, Ly6c2, and associated genes for further analysis.

### Gene ontology analysis highlights differences in response to one and two-component protocols, combining checkpoint inhibitors with ablation or aCD40

**Figure 3:**
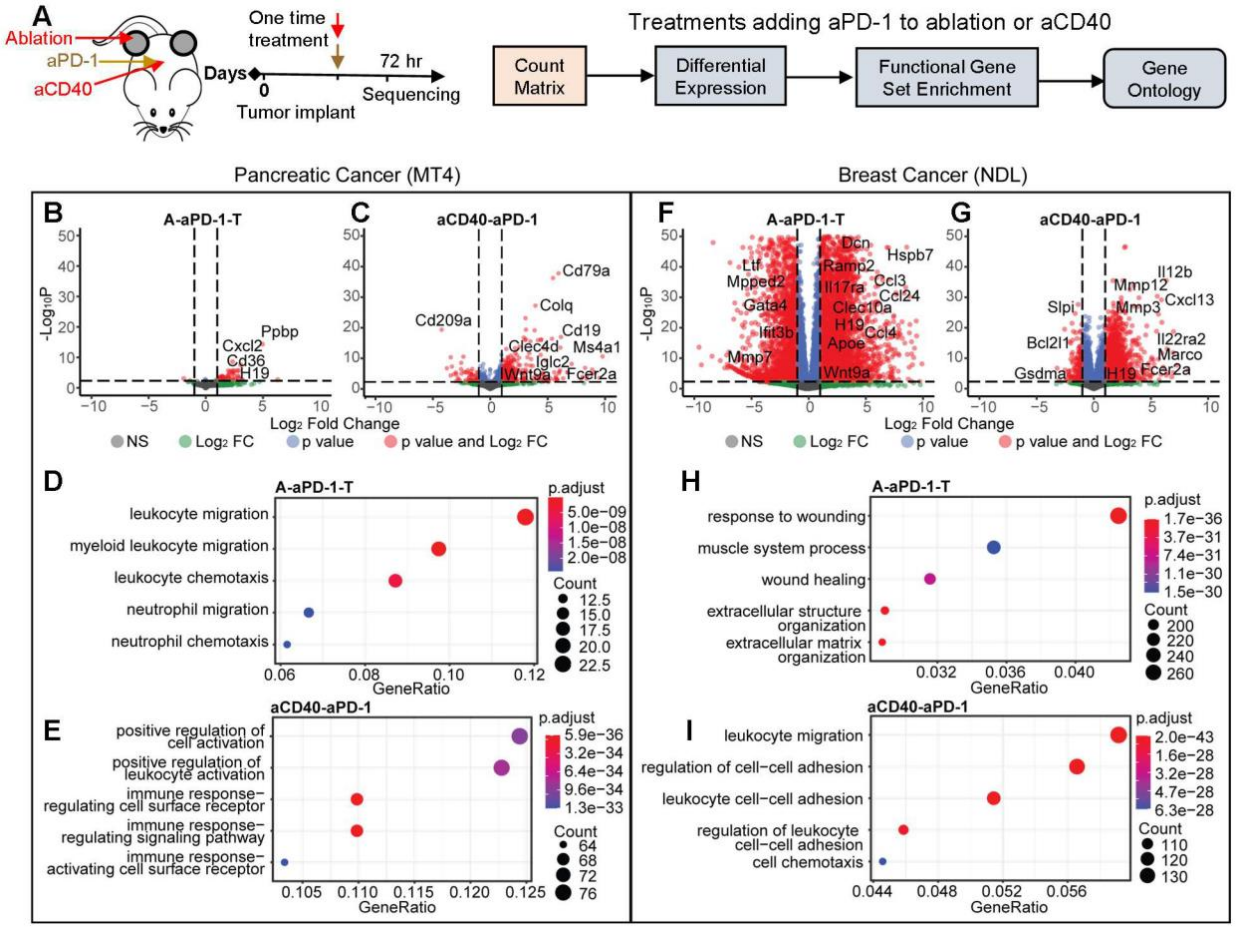
** Ablation or agonist CD40 (aCD40) treatment combined with aPD-1 has reduced, but immune-targeted, effects on gene expression in MT4 pancreatic tumors as compared with highly-differentiated NDL breast tumors.** Two-component treatment protocols of aPD-1 + ablation (n=4, treated tumor, (A-aPD-1-T)) or aCD40 + aPD-1 (n=3 or 4, aCD40-aPD-1) were delivered as a one-time treatment to MT4 (B-E) or NDL (F-I) tumor-bearing mice and compared with a no treatment cohort (n=4). A) Protocol and processing methodology. B-E) Changes in gene expression and ontologies in the MT4 model after (B, D) aPD-1 combined with ablation (A-aPD-1-T) or (C, E) aPD-1 combined with aCD40 (aCD40-aPD-1) treatment. Gene ontologies increased by A-PD-1-T: leukocyte migration and chemotaxis; by aCD40-aPD-1: leukocyte and receptor activation. In the NDL model, aPD-1 was combined with (F, H) ablation or (G, I) aCD40. In the NDL model, gene ontologies increased by A-PD-1-T: wound healing; by aCD40-aPD-1: leukocyte migration, cell adhesion and chemotaxis. Differential expression based on comparison to no treatment control is displayed for an adjusted p value < 0.05 and a fold change > 2, and the changes were subsequently used for gene ontology analysis.

We then applied bulk RNA sequencing to study the impact of single treatments of ablation, aPD-1 or aCD40 (Figure 3A), each applied when tumors reached approximately 4 mm. In total, we acquired 5 data sets in the MT4 model: no treatment, aPD-1 alone, aCD40 + aPD-1, MRgFUS ablation alone, and MRgFUS ablation + aPD-1. We acquired a similar set of data in the NDL model, complementing previously acquired data in this model 26, 27. We found that treatment with aPD-1 alone significantly altered few genes in both cancer models (28 and Figure S3A-B). Here, in the MT4 model, ablation enhanced only the expression of 1 non-annotated gene in the ablated tumor and 0 in the distant tumor by more than 2-fold with an adjusted p value below 0.05. In our previous work with the NDL model, 6406 genes were upregulated by ablation alone in the ablated tumor and 0 in distant tumors, with the change primarily associated with a wound healing inflammatory response 26. Furthermore, ablation increased the secretion of inflammatory cytokines such as IL-6 and Leptin, where the fold change in these markers was greater in the NDL model as compared with the MT4 model (Figure S3C). This demonstrates the significant differences between the inflammatory impact of ablation as a function of the tumor model and suggests there is the potential to combine tumor-debulking ablation with immunotherapy in pancreatic cancer without a large inflammatory impact.

Therefore, we focused the comparative analyses on the treatment combinations. While the untreated tumor cohort increased significantly in diameter over the 72 hrs between treatment and sequencing (Figure S4), in the MT4 model, distant tumor growth was suppressed by the aCD40-aPD-1 or ablation+aPD-1 treatment. In the NDL tumor model, distant tumor growth was reduced for the ablation +aPD-1 treatment compared with the no treatment control and aCD40-aPD-1 cohort (Figure S4).

Evaluation of two-component protocols incorporating aPD-1 together with ablation or aCD40 was then conducted with sequencing performed 72 hours later (Figure 3A). Between the two models, there were significant differences in the genes and pathways affected, particularly by ablation. In the MT4 model, combining aPD-1 either with ablation (Figure 3B) or aCD40 (Figure 3C) enriched immune activation pathways. The combination of ablation + aPD-1 significantly altered the expression of 30 and 50 genes in the treated and distant tumors, respectively (adjusted p value < 0.05 with a fold change greater than 2) whereas aCD40 + aPD-1 significantly altered 557 genes. Genes with greatly enhanced expression as a result of ablation + aPD-1 included Il9r, Il1f9, and Cxcl3, which are associated with immune-related pathways. As a result of aCD40 + aPD-1 treatment, B-cell associated genes, including CD19 and CD79a, were upregulated more than 50-fold. Ablation + aPD-1 enriched immune response pathways associated with myeloid and neutrophil migration and chemotaxis (Figure 3D). aCD40 + aPD-1 enriched pathways associated with cell activation, leukocyte activation, immune signaling pathways and activation of immune cell surface receptors (Figure 3E) based on gene ontology analysis with significantly altered genes.

In the NDL model, the ablation + aPD-1 combination generated an intense wound healing response involving over 8517 genes in the treated tumor (2 genes in the distant tumor) with a fold change greater than 2 and an adjusted p value of less than 0.05 (Figure 3F), including multiple cytokeratins, matrix metalloproteinases and inflammatory cytokines. These findings are consistent with our initial cytokine release analysis of ablation-only treatment (Figure S3C). Similarly, with the aCD40 + aPD-1 combination, the expression of 2858 genes was significantly (adjusted p value < 0.05) altered with a fold change of at least 2 (Figure 3G). Ablation + aPD-1 enriched genes and pathways largely associated with response to wounding, wound healing, and extracellular matrix structure (Figure 3H). Conversely, aCD40 + aPD-1 enriched pathways associated with leukocyte adhesion, migration, and cell chemotaxis (Figure 3I). In our previous work, the addition of TLR9 agonist CpG to ablation similarly reduced the number of upregulated genes and shifted the response from wound healing to an adaptive immune response 26. In summary of the comparative volcano plots, the number and nature of changes in gene expression resulting from combinations of ablation and immunotherapies differed greatly between the breast and pancreatic models.

The gene ontologies associated with the adaptive and innate immune system, TLRs, and known cancer-related genes were then probed in each model based on analysis and clustering using the ablation + aPD-1 treatment or the aCD40 + aPD-1 treatment (Figure S5-6). In each case, the ontologies were clustered for genes that were significantly-enhanced compared to the no treatment control cohort. In the MT4 model, both the ablation + aPD-1 and aCD40 + aPD-1 combination treatments significantly enhanced the innate and adaptive immune response ontology pathways, with upregulation of genes associated with macrophages and B cells (Figure S5). In the NDL model, the ontology pathways associated with the innate and adaptive immune response were not significantly enhanced in the ablation-treated tumors; however, ~100 genes associated with innate and adaptive ontologies were enhanced with aCD40 + aPD-1 treatment (Figure S6). Genes associated with the TLR agonist ontology were enhanced by each treatment. As expected, cancer-related pathways in each model were downregulated by treatment; e.g. Kras was downregulated by aCD40 + aPD-1 in the MT4 model, and Erbb2 (Her2) was downregulated in the NDL breast cancer model with both the ablation + aPD-1 and aCD40 + aPD-1 treatments.

### Digital cytometry provides an overview of changes in immune phenotype

**Figure 4:**
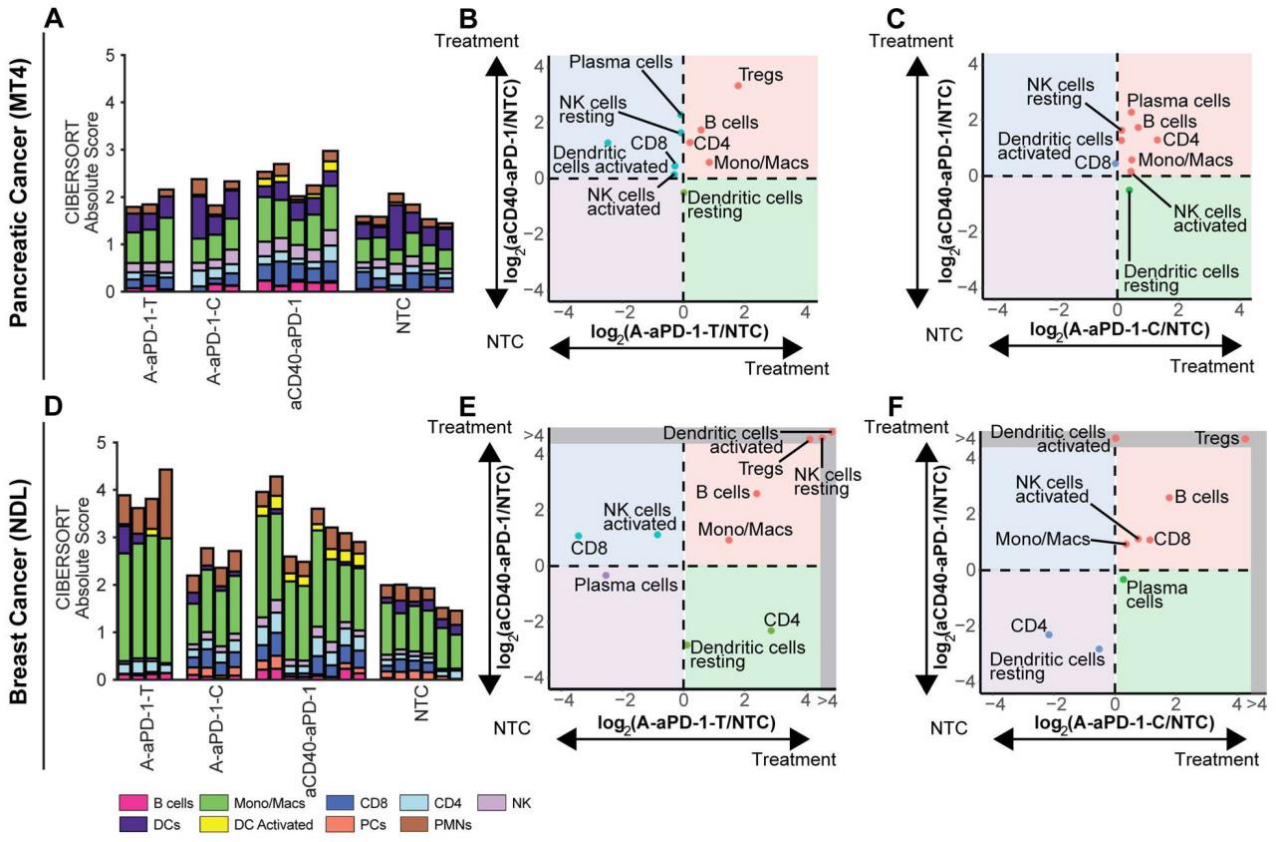
** Comparing digital cytometry results for two-component treatment with aCD40 + aPD-1 or ablation + aPD-1 in the MT4 pancreatic and NDL breast cancer models.** aCD40 + aPD-1 increases leukocytes and activated dendritic cell numbers. Digital cytometry was applied to bulk RNA sequencing data acquired in the MT4 and NDL models under the protocol in Figure 3A. A-C) MT4 model. D-F) NDL model. A, D) CIBERSORTx absolute score. B, E) Fold change from the no treatment control (NTC) cohort, plotted between ablation + aPD-1 in the directly-ablated tumor (A-aPD-1-T) and aCD40 + aPD-1 (aCD40-aPD-1). C, F) Fold change from the NTC cohort, plotted between ablation + aPD-1 in the distant tumor (A-aPD-1-C) and aCD40 + aPD-1 (aCD40-aPD-1). Note that log ratios are based on CIBERSORTx absolute scores. RNAseq experiments were performed with n = 4 replicates with a negative binomial test and Bonferroni correction for p values. Expression of genes in the grey region of E-F was zero in the NTC. Abbreviations: Mast cells (MCs), Polymorphonuclear leukocytes (PMN).

To understand the tumor immune cell composition, we used CIBERSORTx to deconvolve the bulk RNA sequencing data. When we normalized the CIBERSORTx absolute score by the no treatment control cohort for each cell population and compared the log ratio across treatment groups, we found notable immune cell differences between the TME subtypes (Figure 4, Tables S1-2). All treatments elicited a greater response, as measured by the CIBERSORT absolute immune score, in the NDL model (Figure 4D) than in the MT4 model (Figure 4A). A normalization factor calculated from the median expression level of all genes in the signature matrix divided by the median expression level of all genes in the mixture (bulk) under study is applied to all CIBERSORTx outputs in order to generate the absolute score 37. In the MT4 model, monocyte/macrophage populations were significantly expanded by treatment with either aCD40 or ablation combined with aPD-1; additionally, aCD40 + aPD-1 increased tumor-infiltrating activated DCs (Figure 4A-C, Tables S1-2). Ablation + aPD-1 resulted in a trend toward increased regulatory T cells (Tregs) in directly-treated tumors (Figure 4B).

In the NDL breast cancer model, both ablation + aPD-1 and aCD40 + aPD-1 significantly (p < 0.05) expanded monocytes/macrophages and B cells (Figure 4D-F, Tables S1-2). DC activation from ablation + aPD-1 remained localized to the treated tumor (Figure 4E-F), suggesting the absence of a systemic effect with this treatment combination. Ablation + aPD-1 significantly increased CD8^+^ T cells in distant tumors; additionally, aCD40 + aPD-1 treatment also increased CD8^+^ T cells (Figure 4E-F). Tregs were enhanced by the aCD40 + aPD-1 treatment. The results are consistent with gene ontology enrichment analyses with wound healing pathways among the top 5 enriched pathways in the NDL model.

### Modulation of macrophage phenotype for treatment based on naïve TME characterization

Since ablation creates a wound healing response in the NDL TME, we hypothesized that macrophage phenotypes would change according to treatment differences and TME subtypes. Macrophages are often classified by M1 and M2 polarization based on the propensity for nitric oxide (NO) and ornithine production, respectively. Therefore, we specifically looked at macrophage polarization signature genes originally reported from lipopolysaccharide (LPS) stimulation in the context of our treatments 38. Through hierarchical clustering analysis of macrophage signature genes, the macrophage phenotype was not significantly altered by the addition of ablation in the MT4 model (Figure S7). In contrast, we found that in the directly-ablated NDL tumor, the ablation + aPD-1 combination resulted in distinctive transcriptomic upregulation in both M1 and M2 macrophage phenotypes (Figure S7). These changes in gene expression were not observed in the contralateral tumors, reflecting the localized effect of ablation. Thus, the combination treatment of ablation + aPD-1 distinctively altered both M1 and M2 signature gene sets in the ablated NDL tumors, which is consistent with gene ontology enrichment in wound healing pathways.

We further probed the changes in macrophage and monocyte phenotypes in both MT4 (Figure S8) and NDL (Figure S9) cancer models. Based on the LM22M CIBERSORTx signature matrix-derived cell phenotype in the MT4 model, aCD40 + aPD-1 treatment altered genes related to the M1 phenotype and DC activation to a greater extent compared to cells in other myeloid compartments (Figure S8). In the directly-treated NDL tumor, ablation + aPD-1 treatment altered components of the resting macrophage (M0), M1, and M2 phenotypes with a greater Z score compared to aCD40 + aPD-1 treatment, and these components were largely complementary to those modified by aCD40 (Figure S9). Treatment with aCD40 + aPD-1 altered DC activation more prominently than ablation + aPD-1 treatment. Analysis of bulk RNA sequencing data demonstrated that ablation + aPD-1 did not significantly alter genes associated with the monocyte subpopulation phenotype in the MT4 model but significantly upregulated many of the Ccl and Clec family genes in the NDL model (Figure S10A). In contrast, aCD40 + aPD-1 upregulated Ly6c2 in both tumor models (Figure S10A).

Analysis of the long-term survival of NDL tumor mice treated with aCD40 + aPD-1 (Figure S10B) indicated that this treatment alone was effective in this model, whereas preliminary data in the MT4 model (not shown) indicated this was not the case. Therefore, the MT4 treatment strategy was extended to include three and four components and, in particular, aCTLA-4 was added to address immune suppression.

### Extending the treatment protocol to three or four components in the MT4 model of pancreatic cancer

Based on the enhanced effect of aCD40 and the diminished wound healing response in the MT4 pancreatic cancer model compared with the NDL breast cancer model (which is encouraging for the translation of ablation treatment strategies in pancreatic cancer), we then focused on combinations of ablation, aCD40 and checkpoint inhibition in this aggressive pancreatic cancer model. Tumor growth over the 72 hours between treatment and study termination are summarized in Figure S11, confirming reduced growth in distant tumors for all combination ablation-immunotherapy cohorts. We began by applying spectral cytometry to evaluate the effect of aCD40 or aCD40 + checkpoint inhibition on specific immunocytes to first understand the effect of immunotherapy combinations without ablation. We then performed bulk RNA sequencing on subsets of ablation, aCD40 and checkpoint inhibition protocols. We also evaluate survival benefits of these multi-component combinations with ablation.

### Spectral cytometry confirms T-cell and NK-cell activation and differentiates Ly6c populations after aCD40 treatment in the MT4 model

**Figure 5:**
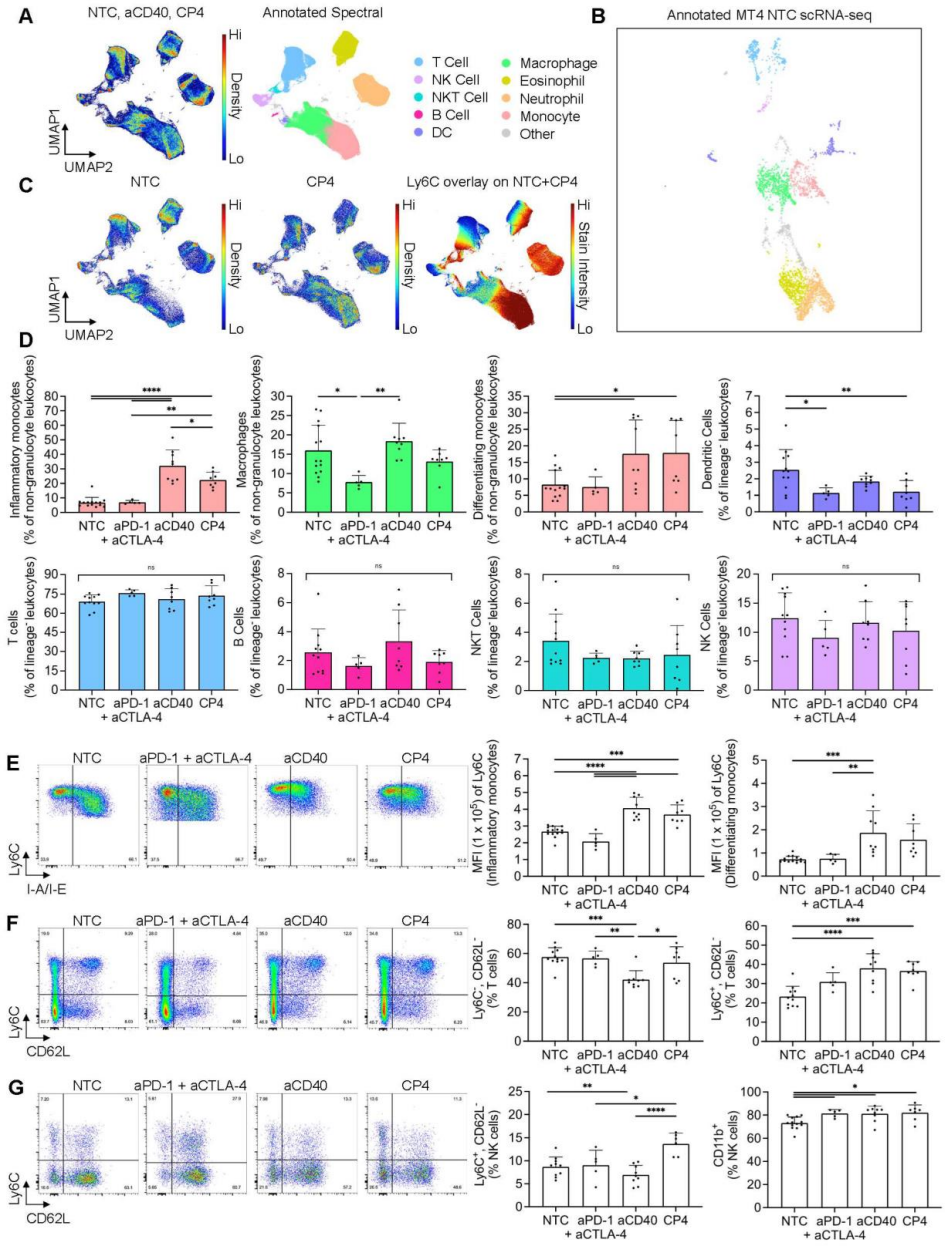
** Applying spectral cytometry to phenotype individual immune cells following treatment combinations of aCD40 and checkpoint inhibitors in the MT4 tumor model revealed mobilized monocytes and increased T-cell and NK-cell effector phenotypes.** MT4 tumor-bearing mice were treated based on the protocol in Figure 3A, comparing a one-time injection of aCD40 to an injection of aCD40 combined with the checkpoint inhibitors aPD-1 and aCTLA-4 (denoted CP4), or aPD-1 and aCTLA-4 alone, using spectral cytometry at 72 hrs. A) Master pseudocolor UMAP and its annotated version (750,000 total events) for the no treatment control (NTC) (n = 5 tumors) (n = 5 tumors), aCD40 (n = 4 tumors), and CP4 treatments (n = 3 tumors) (250,000 events for each treatment evenly distributed among tumor replicates). Combinations of markers used to label each subset are described in the caption for Figure S12A. B) NTC MT4 scRNA-seq plot annotated using a similar number of the same parameters as used in spectral cytometry. C) Pseudocolor UMAP subplots (NTC and CP4 separately) and Ly6C overlay (NTC and CP4 combined), each derived from the master UMAP (250,000 events for each treatment subplot, 500,000 for Ly6C overlay). The colorbar represents Ly6C expression, where red is high and blue is low to zero.D) Major immune subsets as a percentage of non-granulocyte leukocytes (live, CD45^+^Siglec-F^-^Ly6G^-^) or lineage- leukocytes (live, CD45^+^Siglec-F^-^Ly6G^-^CD64^-^). E) Representative pseudocolor dot plots of monocyte populations in response to NTC, aPD-1 + aCTLA-4, aCD40, and CP4 treatment (22,831 events each) with Ly6C - BV605 median fluorescence intensity of each subset. The two monocyte populations were distinguished by I-A/I-E presence (inflammatory monocytes were I-A/I-E^-^ and differentiating monocytes were I-A/I-E^+^) F) T cells (39,220 events each) and G) NK cells (7,971 events each) with respective subsets as a percentage of total T cells or NK cells. The two monocyte populations were differentiated by I-A/I-E presence (inflammatory monocytes were I-A/I-E^-^ and differentiating monocytes were I-A/I-E^+^). Data in D, E, F, and G are presented as mean ± SD. Statistical analyses were performed using one-way ANOVA with Tukey's multiple comparisons test. ns = non-significant, * = p < 0.05, ** = p < 0.01, *** = p < 0.001, **** = p < 0.0001.

After utilizing scRNA-seq and CIBERSORTx methods to deconvolve tumor immune composition, we applied spectral flow cytometry to quantify changes on the single-cell level within MT4 tumors including three or four component protocols and smaller control protocols. We employed a combination of manual gating and unsupervised clustering methods to immunophenotype major subsets of the TME (Figure S12A, B). An equal number of live, CD45^+^ NTC, aCD40, and CP4 events were collected across replicates, concatenated, clustered with UMAP based on fluorescence intensity parameters, and annotated to visualize cell type composition (Figure 5A). To compare these results with scRNA-seq data, we then manually re-annotated the existing NTC MT4 scRNA-seq UMAP clusters using the gene expression of markers profiled via flow cytometry (Figure 5B). Akin to the spectral cytometry UMAP, the scRNA-seq UMAP generally clustered lymphocytes, myeloid cells and granulocytes into three major islands with relative frequencies similar to those found with flow cytometry (Figure 5B).

We then fragmented the overall spectral UMAP into NTC, CP4, and NTC + CP4 subplots to find major differences in leukocyte composition and Ly6C expression after treatment (Figure 5C). Among all populations, we found the greatest differences within the monocyte cluster, as these cells dramatically changed in frequency with aCD40 or CP4 treatment (Figure 5C-D). CD11b^+^CD64^+^Ly6C^+^ monocytes comprised a heterogenous population based on I-A/I-E expression, labeled as I-A/I-E^-^ “inflammatory monocytes” or I-A/I-E^+^ “differentiating monocytes”, as previously described 39. Independent of checkpoint inhibition, aCD40 caused inflammatory monocytes to increase by ~4-5 fold and differentiating monocytes ~2-3 fold, as a percentage of total leukocytes when compared to the NTC cohort (Figure 5D, Figure S12C). Conversely, checkpoint inhibition alone did not alter monocyte frequency or phenotype. To prevent monocyte expansion and granulocyte variance from biasing other frequency analyses across treatments, all other subsets excluding macrophages were analyzed as a percentage of live, CD45^+^CD64^-^Siglec-F^-^Ly6G^-^ cells (lineage^-^ leukocytes). T cells, NKT cells, NK cells, and B cells remained similar in frequency across treatments while macrophages and DCs changed slightly (Figure 5D).

We then further analyzed the monocyte and macrophage compartments to discern phenotypic changes. Qualitatively, we noted a profound difference in the Ly6C, F4/80, and I-A/I-E axes used to immunophenotype these myeloid subsets (Figure 5E, Figure S12D). All monocytes in the aCD40 and CP4 treatment groups upregulated Ly6C and expressed lower levels of I-A/I-E and F4/80 (Figure 5E, Figure S12C-D). Differentiating monocytes found in NTC or aPD-1 + aCTLA-4 mice appeared more committed to a mature macrophage phenotype based on higher F4/80 and I-A/I-E expression as compared to those found in aCD40 or CP4-treated mice (Figure 5E, Figure S12C-D). Taken together, these phenotypic changes suggest that treatments incorporating aCD40 alter the differentiation trajectory of infiltrating, inflammatory monocytes and have downstream effects on macrophage phenotype (Figure S12D) 19.

In addition to monocyte expansion, aCD40 and checkpoint inhibition also altered lymphocyte subsets. As a percentage of total T cells, CD62L^-^Ly6C^+^ T cells expanded with all treatments while CD62L^-^Ly6C^-^ T cells decreased in aCD40 treated animals (Figure 5F). Fluorescence intensity of Ly6C was identical between all CD62L^-^Ly6C^+^ cells across treatments, suggesting these cells were only expanding in the treatment groups rather than obtaining distinct phenotypes as compared to the NTC group (Figure 5F). Ly6C expression on T cells has been associated with homing to lymph nodes and has been positively correlated with IFN-γ, TNF-α, and granzyme B production along with CD4^+^ T cell-mediated cytotoxicity 40. Because CD62L is expressed on naïve subsets, expansion of CD62L^-^Ly6C^+^ T cells is likely representative of an increased frequency of functional effector cells. Beyond T cells, NK subsets also changed in frequency. Ly6C^+^CD62L^-^ NK cells were the most expanded subset in CP4-treated animals compared to all other treatment groups (Figure 5G). Ly6C and CD11b are associated with NK cell maturation and become upregulated during the later stages. By the end of maturation, NK cells are highly cytotoxic but lose proliferative potential and may produce fewer cytokines 41. Consistent with a mature, effector phenotype, we found that CD11b^+^ NK frequencies increased across all treatments (Figure 5G). Together, these results indicate that aCD40 and CP4 treatments shifted the phenotype of monocytes, NK cells, and a subset of Ly6C^+^ effector T cells while the effect of checkpoint inhibition was localized to lymphocyte populations.

To further validate our digital cytometry findings within the DC compartment, we again used flow cytometry to compare the NTC cohort against MRgFUS ablation and CP4 treatment combinations in the MT4 model. In ablated tumors treated with CP4, the percentage of DCs expressing maturation markers CD40, CD80, or CD86 was significantly increased compared to the NTC group (Figure S13A). Contralateral-CP4 tumors, and tumors treated with CP4 only, also had a higher frequency of CD80^+^ DCs compared to the NTC group (Figure S13A). These findings indicate that ablative protocols can synergize with antibody-based immunotherapies to increase DC activation in pancreatic tumors. To evaluate the impact of the different techniques on the major populations estimated by both spectral flow cytometry and CIBERTSORTx are summarized in Figure S13B-E, confirming similar findings for naïve T cells. Differences that likely resulted from mechanical aspects of flow cytometry and bulk RNA sequencing methods are evident in the DC, monocyte, macrophage NK populations (Figure S13D-E).

The most significant results from the spectral cytometry studies are summarized for convenience in Table 1. Most importantly, the effect of aCD40 included changes in the phenotype of T, NK and DC cells to mature, effector and activated phenotypes.

### Bulk sequencing defines changes in gene expression and ontologies for combinations of aCD40, ablation and checkpoint inhibitors in the MT4 model of pancreatic cancer

**Figure 6:**
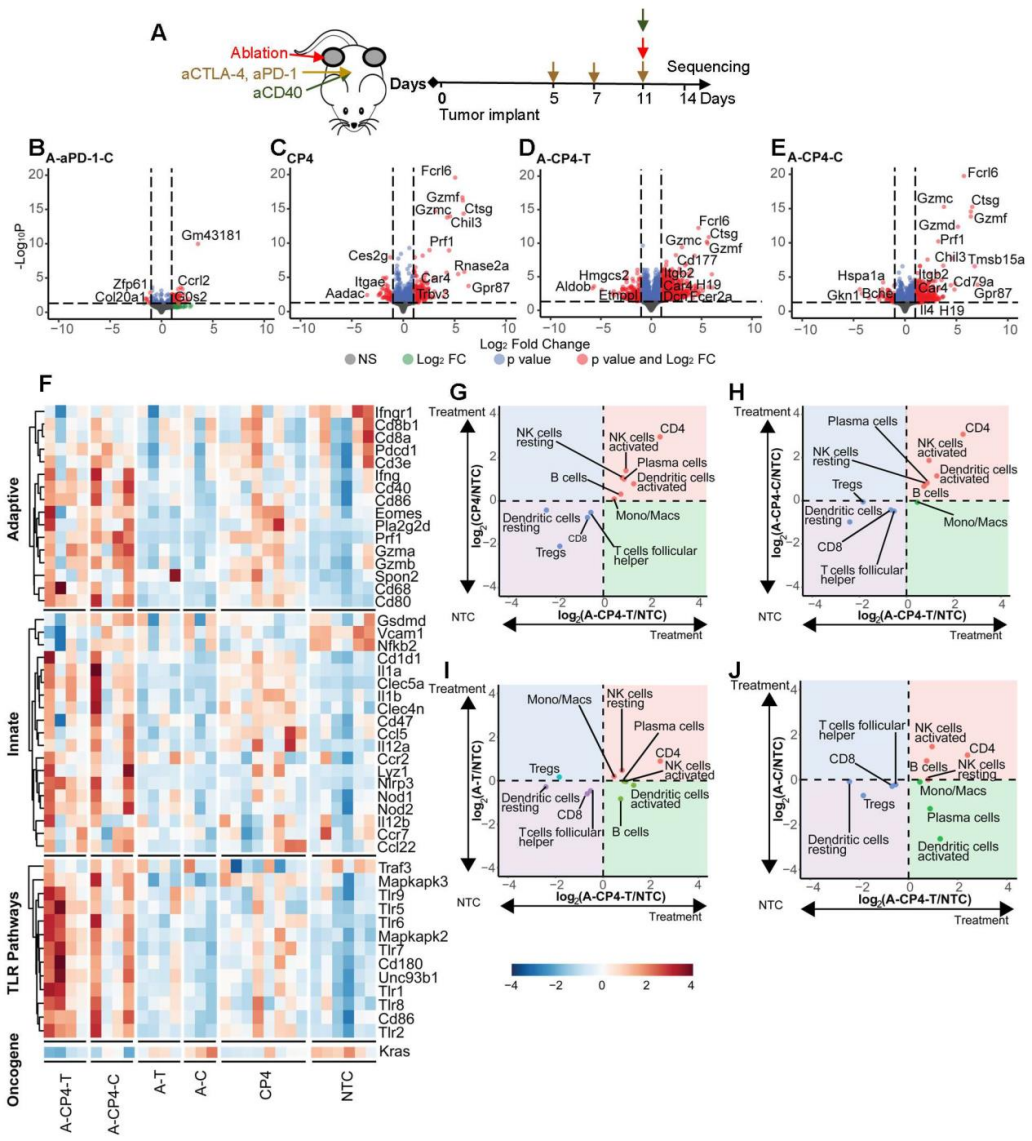
**Digital cytometry analysis in the MT4 model demonstrates the enhanced immune activation resulting from a four-component treatment combining ablation with CP4 (aCD40 + aPD-1 + aCTLA-4).** A) Treatment protocol. Mice were treated with two doses of checkpoint inhibition priming prior to an application of checkpoint inhibitors with aCD40 and ablation each added in a subset of mice (n=4 each group) and compared to no treatment control (NTC) mice (n=4). Bulk RNA sequencing was performed 72 hrs after ablation. B-E) Volcano plots showing gene expression response to treatment combinations. B) Ablation + aPD-1 in the distant tumor (A-aPD-1-C) altered expression of 50 genes. C) CP4 altered expression of 285 genes. D-E) Ablation + CP4 resulted in D) 1379 differentially expressed genes in the treated (A-CP4-T) tumor and E) 475 differentially expressed genes in the distant (A-CP4-C) tumor. F) Ablation + CP4 upregulated genes in key immune pathways such as the adaptive immune (GO:0002819), innate immune (GO:0045088) and toll-like receptor (TLR) (GO:0002224) pathways and downregulated the Kras cancer gene in both the treated and contralateral tumors to a greater degree than systemic CP4 treatment alone. G-J) Digital cytometry was applied to bulk RNA sequencing data. Fold change from the NTC is plotted between ablation + CP4 in the ablated tumor (A-CP4-T) versus G) CP4, H) ablation + CP4 in the distant tumor (A-CP4-C), I) ablation-only in the treated tumor (A-T), and J) ablation-only in the distant tumor (A-C). Ablation + CP4 stimulated immune cell changes in both the treated and distant tumor sites, increasing CD4^+^ T cells and dendritic cell and NK-cell activation.

We then compared the results of the two-component treatment with three or four-component treatment in the MT4 model, based on tumor bulk RNA sequencing data (Figure 6A). To investigate the systemic effect of ablation + aPD-1, we explored the differentially expressed genes in the distant tumor and found ablation with aPD-1 altered expression of 50 genes (Figure 6B). CP4 treatment (Figure 6C) significantly altered 285 genes with a fold change greater than 2. In contrast, ablation combined with CP4 resulted in 1379 significantly differentially expressed genes in the directly-treated tumor (Figure 6D) and 475 significantly differentially expressed genes in the distant tumor (Figure 6E), with particular activation of granzyme-related genes. The genes altered by ablation + CP4 in the treated tumor were enriched in leukocyte migration, chemotaxis processes, and myeloid leukocyte and neutrophil migrations (Figure S14A).

Based on gene ontology analysis, ablation + CP4 further upregulated genes in key immune pathways such as the adaptive, innate, and TLR pathways and downregulated other cancer genes in both the treated and distant tumors to a greater degree than systemic CP4 treatment alone (Figure 6F). Through CIBERSORTx analysis of bulk RNA sequencing data 9, we compared immune cell responses from different treatments log-normalized to the NTC groups, with P values summarized in Table S3. CP4 treatment alone significantly enhanced CD4^+^ T cells in the bilateral tumors with a trend toward increased resting and activated NK cells and plasma cells (Figure 6G). The combination of ablation + CP4 similarly increased CD4^+^ T cells, and activated NK cells (Figure 6G-H). In contrast, ablation alone did not expand specific immune subsets (Figure 6I-J). Similar to the results reported for spectral cytometry, CP4 treatment and ablation + CP4 increased activated DC populations in both treated and distant tumors although the change was not significant for the CIBERTSORTx analysis (Figure 6G-H, Figure S13A, Figure S14B).

In addition, CP4 with and without ablation upregulated monocyte/macrophage and granzyme-associated genes (Figure S15). In particular, the addition of ablation to CP4 immunotherapy significantly increased IL-6, Adgre1, Cd64, Ly6a2 and Ly6c2 in the MT4 pancreatic tumors (Figure S15A-E), indicating a population of monocytes and macrophages was particularly enhanced by this combination treatment. Among the genes with the greatest fold change resulting from the CP4 treatment in pancreatic cancer were granzymes, Ctsg and Prf1 (Figure S15F-N). Granzymes are serine proteases released by cytoplasmic granules within monocytes, cytotoxic T cells, and NK cells. In evaluating the expression of Gzmg across treatments producing a significant increase in this granzyme compared to the NTC cohort (Figure S15M), only a small increase was observed with aPD-1 treatment alone (~6-fold), likely resulting from changes in T-cell or NK-cell phenotype. The Gmzg enhancement increased to 8-fold for the ablation + aCD40 + aPD-1 combination, 59-fold for CP4 treatment and 96-fold for ablation + CP4 treatment in the distant tumor. Ctsg encodes for a member of the peptidase S1 protein family, which is typically found in granules of polymorphonuclear leukocytes. Changes in Ctsg were significant only for treatment combinations including ablation and immunotherapy or CP4 and reached 110-fold in distant tumors for mice treated with ablation + CP4 (Figure S15N). In addition, we find that matrix metalloprotease expression (e.g. MMP8) is significantly enhanced with the immunotherapy treatments including aPD-1 and aCD40 and the enhanced expression is maintained in the distant tumor for the combination of ablation and aPD-1 or CP4 (Figure S15O).

### Combination treatments reduce tumor growth and enhance survival in the MT4 cancer model

**Figure 7:**
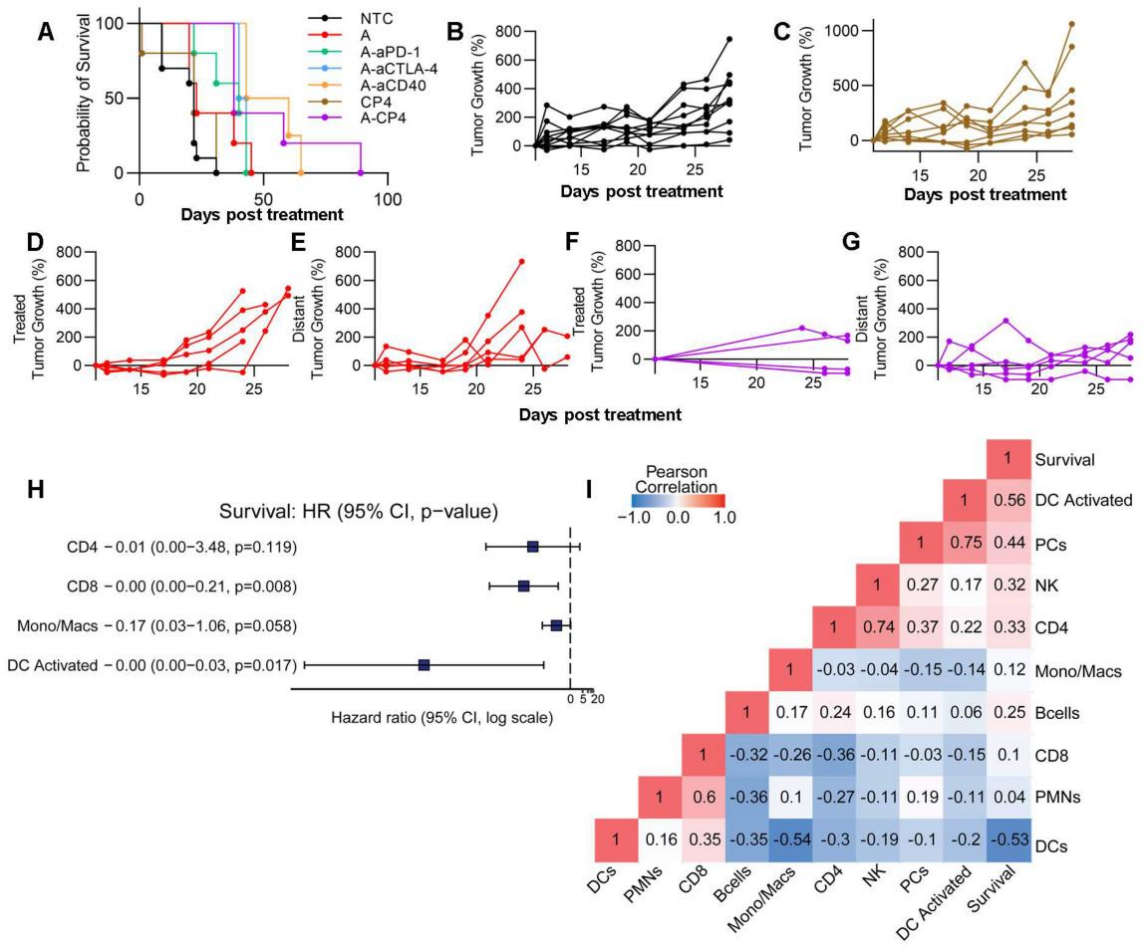
** Combination of ablation with CP4 in the MT4 tumor model generates a systemic anti-tumor effect.** Tumor growth from the protocols shown in Figure 6A (n=4 for treatments and n=3 for the no treatment control (NTC) cohort). A) Survival for NTC, Ablation alone, Ablation + aPD-1, Ablation + aCTLA-4, Ablation + aCD40, CP4 alone, Ablation + CP4 cohorts. B-G) Tumor growth for B) NTC, C) CP4, D) Ablation in the treated tumor, E) Ablation in the distant tumor, F-G) Ablation + CP4 in the treated (F) and distant (G) tumor. Tumor volume plots are provided in Figure S16. H) Cox-hazard analysis comparing cells to survival outlined the importance of activated dendritic cells. I) Pearson correlation analysis indicated high correlations between 1) survival and dendritic cell (DC) activation, 2) NK cells and CD4^+^ T cells and 3) plasma cells (PCs) and dendritic cell activation.

Finally, we conducted an evaluation of survival and tumor growth after a single treatment cycle. When combining MRgFUS ablation with CP4, there was a significant reduction in tumor growth (p < 0.0001, ANOVA) and a factor of 3 extension in survival (p < 0.0001, log rank test) as compared with immunotherapy alone (Figure 7A). We found that the CP4 and ablation regimens alone did not significantly alter tumor growth in the MT4 model (Figure 7C-E), but the addition of CP4 with ablation significantly reduced tumor growth over 17 days after treatment (Figure 7F-G, Figure S16). Since multi-component combinations induce multiple immune pathways and stimulate immune cells, we performed a Cox proportional hazards regression analysis based on selected CIBERSORTx cell populations (CD4^+^, CD8^+^, Mono/Macs, activated DCs, selected based on preliminary results) and found that activated DCs were most important in determining survival outcome (Figure 7H). With Pearson correlation analysis, we found that activated DCs, NK cells, and CD4^+^ T cells positively correlated with longer survival duration in the MT4 model (Figure 7I). Further, CD4^+^ T cells, plasma cells (PCs), NK cells and DC activation all positively correlated with one another (Figure 7I).

Taken together, the spectral cytometry data suggest that Ly6c2 expression is enhanced on multiple cell types as a result of CP4 treatment, and bulk sequencing indicates that the Ly6c2^+^ population is further enhanced with the addition of ablation. Further, activated immune cell populations were detected in both treated and contralateral tumors following treatment with ablation + CP4, and the results point towards enhanced DC and NK activation with greater numbers of CD4^+^ T cells due to this treatment.

## Discussion

In this study, we set out to evaluate the differential impact of combinatorial ablation and immunotherapy in mouse models of cancer and to develop tools to assess multi-component protocols. We evaluated the effects of treatment in diverse TME subtypes that are representative of human cancers undergoing trials with MRgFUS ablation: 1) a pancreatic tumor model with a dense stroma and sparse tumor cell distribution (based on the KPC model), and 2) an Her2^+^ epithelial breast tumor model (based on the PyMT model) with a dense tumor cell distribution and larger number of immune cells. In human trials of MRgFUS in metastatic cancers, the ultimate goal is to ablate a local region while inducing an anti-tumor immune response to treat the systemic disease. We seek to assess both the local and distant effect of treatment here.

We summarize the major results from the study in Table 2, demonstrating differences in gene expression, leukocyte populations and survival across breast and pancreatic cancer and treatment types. We organize the discussion to consider the methodologies, the effect of ablation and finally the impact of immunotherapy components. Our results point to very significant differences in the impact of thermal ablation across models and support combining ablation with antibody-based immunotherapy (particularly in pancreatic cancer). Ultimately, we found that the combination of ablation and immunotherapy based on checkpoint inhibitors and aCD40 enhanced survival in pancreatic cancer as compared with immunotherapy alone. This suggests that strategies to disrupt the TME stroma and promote restructuring of the microenvironment could be beneficial when combined with antibody-based immunotherapy in future translational studies. Alternatively, the inflammatory effect of ablation must be considered when designing treatment protocols in Her2^+^ breast adenocarcinoma.

### Study methodology and resulting insights

From bulk sequencing, we learned that the CP4 immunotherapy treatment enhanced perforin, granzyme and Ctsg related genes and this effect was further increased with the ablation-CP4 combination (with fold changes as high as 100-fold) likely contributing to the therapeutic response. This enhancement results in part from the changes in the NK and T-cell phenotypes (as confirmed by the small increase observed with aPD-1 treatment alone). From digital cytometry based on bulk RNA sequencing, we learned that this ablation-CP4 combinatorial therapy enhanced a combination of DCs, NK and CD4^+^ T cells. Bulk sequencing also pointed to the enhanced MMP expression in response to these treatments, where MMPs can reduce the tumor stroma.

The established LM22M CIBERSORTx signature does not specifically address the Ly6c phenotype resulting from the treatments applied here and could not fully address the individual cell phenotype. Therefore, we augmented this method with spectral cytometry, evaluating checkpoint inhibition, aCD40, and CP4 treatment. In particular, spectral cytometry pointed to a shift in T-cell, NK-cell, DCs and monocyte phenotype when analyzed as a function of change on a per-cell basis. This is consistent with studies indicating aCD40 stimulation drives T-cell activation independent of TLR pathways and with the existing literature regarding checkpoint expression on T cells and NK cells 14, 42. Taken together, these results show that combinatorial immunotherapy elicits broad changes in immune cell phenotype and suggests that the perforin/granzyme enhancement is found in multiple cell types.

### The impact of ablation on response across breast and pancreatic cancer

MRgFUS ablation debulks tumor with spatial and dose precision and also stimulates ICD, DAMP production and immune cell infiltration in mouse tumor models 27-30. In the context of diverse TME subtypes 5, MRgFUS ablation is notable because it can precisely disrupt the TME, facilitating the tailoring of immunotherapy combinations specific to particular TME subtypes. Thermal ablation has been broadly applied in clinical management, where advantages of thermal ablation include the ability to monitor temperature with MRI, the opportunity to heat fix the poorly perfused tumor core while releasing antigen from the tumor rim, and the increase in perfusion in the treatment rim with enhanced therapeutic delivery 43. Histotripsy is an alternative technique that uses a high mechanical index, and when combined with immunotherapy in poorly immunogenic melanoma tumor models produced M1 polarization and increased CD8^+^ T cells in directly-treated tumors 44. An advantage of histotripsy is that tumor antigen can be released from the entire tumor without heat-based denaturing.

Compared to murine NDL tumors, our initial single-cell sequencing and histological characterization of treatment-naïve murine MT4 tumors demonstrated lower proportions of lymphocytes and myeloid cells, both important in the anti-tumor cancer immunity cycle 45, a finding similar to clinical characterization of human PDAC 6. Immunohistochemistry demonstrated the significant difference in tumor cell density between the two tumor models as previously described for human tumors.

We sought to demonstrate that there are profound differences in gene expression based on ablation-alone across the breast and pancreatic cancer models and likely between ablation in human breast and pancreatic cancer. At 72 hours after a partial ablation, thousands of genes were altered with an adjusted p value < 0.05 and a fold change > 2 by ablation alone in breast cancer and few genes in pancreatic cancer (Figure 3 and Table 2). We have previously demonstrated the inflammatory response that results from ablation in the NDL model 26, 27. In the NDL model, focused ablation and aPD-1 enriched wound healing pathways and modulated the macrophage phenotype. The intense inflammatory and wound healing response in the NDL model may result from the impact of thermal ablation on the dense tumor cell network and from the ablation of fat cells. In fact, ablation of the NDL tumor combined with aPD-1 resulted in a 12-fold increase in Il6 gene expression 26. We also assayed blood cytokines at 5 hours after ablation and the fold changes in IL6 and LEPTIN were greater in the NDL than MT4 model. The impact of tumor cell death may be smaller in the MT4 model, in part, due to the sparse nature of the tumor cell organization. In the NDL model, we previously found that immune priming before ablation improved outcomes when compared to a non-priming treatment protocol 28, and this may result, in part, from mitigating the wound-healing response. We cannot rule out an impact of the differing immune environment, as documented in Figure S1, in the inflammatory response, and differentiating these effects will be the subject of future work. We hypothesize that a very dense epithelial human tumor (such as the NDL model) will produce a greater wound healing response from thermal ablation, and this can impact the response to immunotherapy.

We explored a range of treatments in the pancreatic MT4 model spanning ablation alone, aPD-1 alone, aCD40 + aPD-1, aCD40 + aPD-1 + aCTLA-4 (denoted CP4), ablation combined with aPD-1, and ablation combined with CP4. In each of these scenarios, the gene ontologies resulting from MT4 treatment were dominated by immune-related ontology pathways rather than a wound healing response. Focused ablation combined with aPD-1, aCD40, or CP4 extended survival by up to 3-fold in the MT4 model. Immunotherapy alone did not enhance survival, possibly due to the lower lymphocyte fraction and dense stroma that limits diffusion of the antibodies within the tumor, suggesting that debulking may be particularly important in pancreatic cancer.

### Impact of aCD40, aPD-1 and aCTLA-4

While focused ultrasound ablation is safe and effective at tumor debulking, when combined with systemic aPD-1 therapy, DCs were activated only in locally-treated tumors. We reasoned that TME disruption and subsequent stimulation of durable anti-tumor immunity with memory required the activation of interconnected key players along the adaptive immunity axis. aCD40 activates DCs via aCD40 receptor ligation 17 while, to a lesser extent, focused ablation can also activate DCs as part of the adaptive immune response pathway to external stress 30. Here, systemic administration of aCD40 along with aPD-1 activated DCs in both tumor models. aCD40 ligation has also been reported to increase the infiltration of Ly6c^+^ expressing monocytes/macrophages while prolonging activity of T cells 46, forming a rationale for combining the aCD40 agonist with ICI treatments. aCD40 further remodeled the myeloid population towards Ly6c^+^ monocytes/macrophages. aCD40 + aPD-1 enhanced Ly6c expression by approximately 3-fold and 4-fold in the MT4 and NDL models, respectively, and this effect was enhanced by the incorporation of thermal ablation. We found that the enhanced Ly6c expression included changes in monocytes but also in lymphocyte populations. To successfully treat immune-cell-excluded tumors such as pancreatic cancer, thermal ablation synergizes with the combination of immune therapies. Because of the inherently inflammatory effect of ablation, treatment combinations addressing both myeloid and lymphocyte populations may be required.

Further work is required to fully understand the impact of checkpoint inhibitors in the response achieved here. aPD-1 alone enhanced Gzmg expression 6-fold. When both checkpoint inhibitors were added to aCD40 treatment, Gzmg expression increased 59-fold. Gzmg expression increased further with the addition of ablation to this protocol. Spectral cytometry was applied to compare the CP4 vs aCD40 treatment to assess the impact of checkpoint inhibitors and indicated that both T-cell and NK-cell phenotypes were impacted by the CP4 treatment. Both cell types are known to express the PD-1 and CTLA-4 checkpoints 47.

### Study limitations

The use of aCD40 treatment in mice is compromised by the rat IgG background of the available antibodies 48. As a result, the study was limited to a single injection of the aCD40 antibody. Multiple injections have been shown to result in liver accumulation of the 2^nd^ dose 48. We included a subset of all possible combinations. Further work is required to fully characterize all possible parameters and combinations.

### Summary

The bridging role of aCD40 agonists within the tumor immunity axis inspired us to combine ablation with CP4 to explore the effects of aCD40 synergy with lymphocytes, the myeloid compartment and stromal disruption. We set out to explore the use of TME subtypes to guide a cancer treatment design built around an MRgFUS ablation tumor debulking strategy and discovered that, combining MRgFUS ablation with non-redundant immune cell activation, we can generate a systemic immune response and enhance survival in a pancreatic cancer model.

## Methods

Additional methods are available online in the Supplementary Methods and Materials sections.

### Ethics Statement

All experiments and methods were performed in accordance with relevant guidelines and regulations. Specifically, all animal experiments were conducted with approval from the Stanford University Administrative Panel on Laboratory Animal Care (APLAC).

### Animal Models and Cell Lines

The murine *neu* deletion (NDL) metastatic mammary adenocarcinoma cell line was obtained from the Alexander Borowsky Laboratory (UC Davis, Davis, CA). Four-week-old FVB/n female mice, purchased from Charles River (Wilmington, MA), were transplanted with NDL tumor biopsies (~1 mm^3^) bilaterally into the fourth and ninth inguinal mammary fat pads. 15 days later, when tumors reached ~4 mm in longest dimension, mice were randomized into treatment groups. Mice were euthanized when the tumor volume reached the humane endpoint.

The murine MT4 (Kras^+/LSL-G12D^; Trp53^+/LSL-R172H^; Pdx1-Cre) metastatic pancreatic cancer cell line was obtained from Dr. David Tuveson (Cold Spring Harbor Laboratory Cancer Center, Cold Spring Harbor, NY). Four-week-old C57BL/6 female mice were purchased from Charles River (Wilmington, MA) and subcutaneously injected with 4 x 10^5^ MT4 cells in 40 μL of 1:1 PBS -/- and Matrigel (356234, Corning) bilaterally in the hind flank. Treatment commenced on day 5 after tumor inoculation.

### Therapeutic and sequencing protocols

A total of 85 mice were studied. Animals bearing MT4 tumors received three doses of checkpoint inhibitors as follows: 200 μg anti-PD-1 and 200 μg anti-CTLA-4 injected intraperitoneally (i.p.) on days 5, 7 and 11; 100 μg aCD40 was administered on day 11; in cohorts receiving ablation, ablation was performed on day 11. Animals bearing NDL tumors received one dose of each checkpoint inhibitor and aCD40 on day 15. Ablation was performed on day 15 in NDL-bearing animal cohorts that received ablation. RNA sequencing and immunohistochemistry were both performed 72 hours after the final treatment(s) (day 14 for MT4 tumor-bearing animals and day 18 for NDL tumor-bearing animals). Tumor size was measured twice weekly in survival cohorts.

### MRgFUS ablation protocol

MRgFUS parameters were constant across TME subtypes and immunomodulators were modified based on the TME phenotypes. Our previous work has demonstrated that a peak temperature range of 65 to 70 °C improves thermal ablation outcomes by avoiding tissue boiling and minimizing the heat-fixed volumes 26-28.

All ablations were performed under MR guidance on a Bruker BioSpec 7T small animal MR system (Bruker Biospin) with core body temperature monitoring using a 16-element annular array transducer operating at 3 MHz (Imasonic SAS) 49. Acoustic pressure was calibrated with a fiber optic hydrophone (HFO690, Onda Corp.) in a degassed water tank under free-field conditions. Prior to ablation, mice were given 0.05-0.1 mg/kg buprenorphine subcutaneously (s.c.) and 0.1-0.5 mmol/kg gadoteridol (Bracco Imaging) intraperitoneally and imaged with a T1w RARE (TE/TR = 10/700 ms, FOV = 4.8 cm x 4.8 cm, MTX = 196 x 196, ST/SI = 1/1 mm, 19 slices) sequence for tumor localization and treatment planning. Tumors were then ablated and temperature was monitored in real time via the MR proton resonance frequency shift using Thermoguide Software (Image Guided Therapy), with α = -0.0101 ppm/ºC, TE/TR = 4.5/21 ms 26.

Continuous wave (CW) insonation was employed at 3.1 MPa in a circular pattern (diameter of 2 mm, scan speed of 1 revolution per second) until the targeted volume reached at least 60 ºC and a thermal dose in cumulative equivalent minutes at 43 degrees (CEM43) of more than 5000 was achieved 26.

### RNA seq analysis

For single-cell sequencing analysis, we built a pipeline around the Seurat package to process the 10x sequencing output results 50. Briefly, we first filtered for mitochondrial percentages and regressed around the mitochondrial variations. We then integrated the MT4 and NDL tumor datasets together with Seurat integration function 34. Principal component analysis (PCA) was performed on the integrated datasets and we used the elbow plot method to determine the optimal component (75) for downstream analysis. To determine the optimal parameters for k nearest neighbors, resolution and prune number, we sought to minimize within-cluster variance based on Euclidean distances. We constructed a hyperparameter grid of k nearest neighbors (k = 2, 5, 10, 15, 20, 25, 30), resolution (0.2, 0.4, 0.6, 0.8, 1, 1.2), and prune number (0, 0.001, 0.0015, 0.002, 0.0025, 0.003, 0.0035) to iteratively re-cluster for all combinations of parameters. We used the optimal parameters (k = 20, res = 0.4, prune = 0.0015) to generate UMAP clustering with Seurat built-in functions. To annotate the clusters, we first extracted the top 20 most differentially expressed genes from each cluster (as compared with all other clusters) and compared them to cell canonical gene markers and markers from our spectral cytometry panel to annotate the clusters.

### Reagents

The checkpoint inhibitors, anti-mouse PD-1 antibody (rat IgG2a, clone RMP1-14) and anti-mouse CTLA-4 antibody (mouse IgG2b, clone 9D9) as well as agonist mouse anti-CD40 (rat IgG2a, clone FGK4.5/FGK45) were purchased from Bio X Cell (West Lebanon, NH). Flow cytometry reagents can be found in the Supplementary Methods.

### Statistical Analysis

Statistical analyses were performed using GraphPad Prism 8 software (GraphPad Software Inc). Results are presented as mean ± SD, unless otherwise indicated. One-way ANOVA was performed for all analyses of three or more groups followed with Tukey's correction for multiple hypotheses in GraphPad Prism. Analysis of differences between two groups was performed using an unpaired t-test assuming unequal variance. P values less than 0.05 were considered significant.

## Supplementary Material

Supplementary methods, figures, and tables.

## Figures and Tables

**Table 1:**
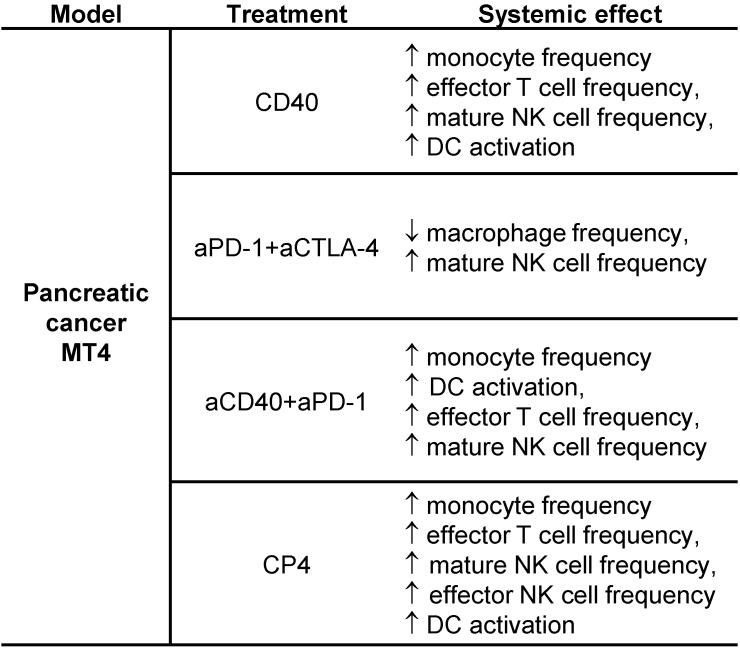
Summary results for spectral cytometry studies in the MT4 tumor model.

Treatment protocols including CD40 alone, aPD-1 + aCTLA-4, aCD40 + aPD-1, and aCD40 + aPD-1 + aCTLA-4 (denoted CP4) were studied. Most importantly, the effect of aCD40, as determined in the lower 2 protocols, included changes in the phenotype of T cells, NK cells and DCs to mature, effector or activated phenotypes.

**Table 2:**
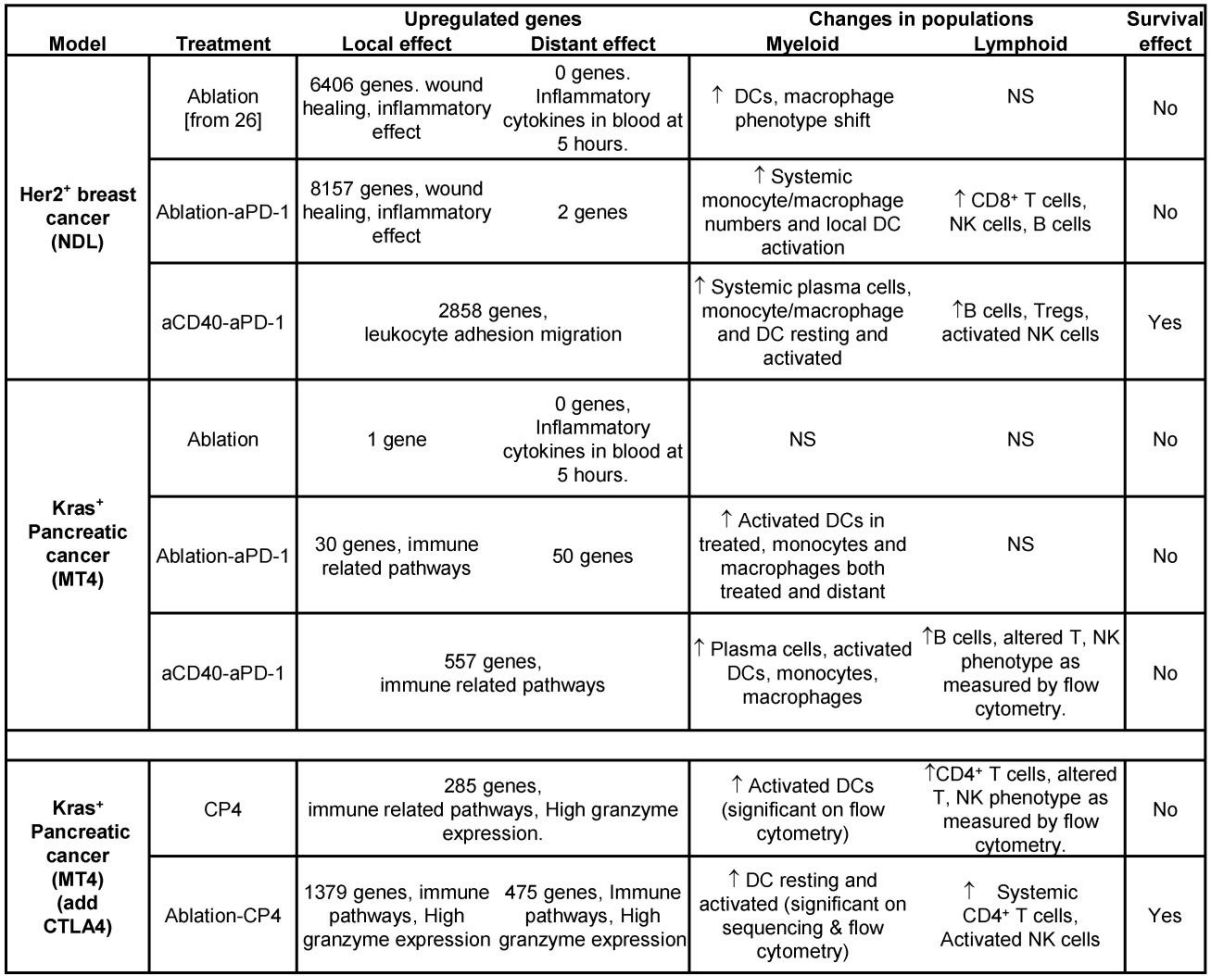
Summary table.

(Top rows) Comparing the MT4 and NDL tumor models across protocols of ablation alone, ablation + aPD-1 and aCD40 + aPD-1. Findings at 72 hours after ablation: 1) the number of genes altered by each individual treatment (for an adjusted p value < 0.05 and a fold change > 2) was greater in the NDL model compared to the MT4 model, 2) ablation created a profound wound healing response in the dense Her2^+^ breast tumors, 3) adding aCD40 to the protocol enhanced B-cell numbers in both models, 4) enhanced survival was achieved in the NDL (but not MT4) model with a simple 2-component treatment (aCD40 + aPD-1). (Bottom rows) Summary table for the MT4 model when treated with either aCD40 + aPD-1 + aCTLA-4 (denoted CP4) or ablation + CP4. Adding aCTLA4 to the treatment enhanced: 1) immune response as particularly noted in the expression of granzyme genes, 2) lymphocyte maturity or activation, as assessed by flow cytometry, 3) survival. NS = Not Significant.

## Abbreviations

aCD40: anti-cluster of differentiation 40
aCTLA-4: anti-cytotoxic T-lymphocyte-associated protein 4
aPD-1: anti-programed cell death protein-1
CD: cluster of differentiation
CEM: cumulative equivalent minutes
CP4: aCD40 + aPD-1 + aCTLA-4
CW: continuous wave
DAMP: damage associated molecular pattern
DC: dendritic cell
FACS: fluorescently activated cell sorting
GEP: gene expression profiles
H&E: hematoxylin and eosin
ICD: immunogenic cell death
ICI: immune checkpoint inhibitors
KPC: Kras^+/LSL-G12D^, Trp53^+/LSL-R172H^, Pdx1-Cre
LM22: leukocyte signature matrix of 22 cell types
LM22M: mouse version of LM22
LPS: lipopolysaccharide
MRgFUS: magnetic resonance guided focused ultrasound
MT4: murine pancreatic adenocarcinoma
NDL: Her2^+^, Ki67^+^, ER/PR negative with neu deletion
NK: natural killer
NO: nitric oxide
NTC: no treatment control
PC: plasma cell
PCA: principle component analysis
PDAC: pancreatic ductal adenocarcinoma
pDC: plasmacytoid dendritic cells
PMN: polymorphonuclear leukocytes
scRNA-seq: single cell RNA sequencing
TLR: toll-like receptor
TME: tumor microenvironment
UMAP: uniform manifold approximation and projection
