# Supplementary material for: Multiomic analysis for optimization of combined focal and immunotherapy protocols in murine pancreatic cancer
Source: Theranostics. 2022 Nov 14;12(18):7884–902. doi: 10.7150/thno.73218 (PMC9706583; doi:10.7150/thno.73218)
Supplement: Supplementary file 1 — Supplementary methods, figures, and tables. [file thnov12p7884s1.pdf]

Multiomic analysis for optimization of combined focal and immunotherapy protocols in murine pancreatic cancer

James Wang, Brett Z. Fite, Aris Kare, Bo Wu, Marina Raie, Spencer K. Tumbale, Nisi Zhang, Ryan R. Davis, Clifford G. Tepper, Sharon Aviran, Aaron Newman, Daniel A King, Katherine W. Ferrara

Supplementary Methods

RNA seq tissue preparation and CIBERSORTx protocol

For bulk sequencing analysis, tumors were collected and flash-frozen 3 days after treatment for RNA sequencing at the UC Davis Comprehensive Cancer Center's Genomics Shared Resource following the same protocol as previously published [1]. Briefly, cellular RNA was isolated, extracted and purified for next generation sequencing. RNA-seq raw data was processed with the STAR-StringTie-Cufflinks pipeline and mapped with the reference mouse genome assembly (GENCODE, GRCm38, release 05/2017). We built a pipeline around clusterProfiler package to perform gene ontology analysis analysis [2] and summarized our results with volcano plots and heatmaps [3,4]. To determine cell proportions, we used CIBERSORTx to deconvolve bulk sequencing RNA data [5]. From the CIBERSORTx output, we further compared cell proportions to each treatment by plotting the log-transformed normalization of cells from each treatment against each other.

For deconvolution of bulk RNA seq data, we used an LM22 signature matrix adapted for mouse. LM22 is a gene-expression signature matrix built using expression data from 547 genes, to distinguish 22 immune cell populations. Originally adopted for deconvolution of human immune cell types, the LM22 matrix was transposed into a mouse LM22 matrix by mapping human gene names to orthologous mouse genes. Gene expression levels were summarized as transcripts per million and then used as input for CIBERSORTx deconvolution with B-mode batch correction using the web application for CIBERSORTx (cibersortx.stanford.edu).

Flow Cytometry Antibodies:

Pacific Blue rat anti-mouse CD45 (30-F11) and PerCP rat anti-mouse I-A/I-E (M5/114.15.2) were purchased from BioLegend. BUV496 Armenian hamster anti-mouse CD3e (145-2C11), BUV563 rat anti-mouse CD115 (T38-320), BUV615 rat anti-mouse Ly-6G (1A8), BUV661 rat anti-mouse CD11b (M1/70), BV605 rat anti-mouse Ly-6C (AL-21), BV650 rat anti-mouse CD62L (MEL-14), BV750 rat anti-mouse CD117 (2B8), BV786 rat anti-mouse CD19 (1D3), BB700 mouse anti-mouse NK1.1 (PK136), PE rat anti-mouse Siglec-F (E50-2440), and rat anti-mouse CD16/CD32 (2.4G2) were purchased from BD Biosciences. eFluor450 rat anti-mouse F4/80 (BM8), eFluor506 Armenian hamster anti-mouse CD11c (N418), Alexa Fluor 532 rat anti-mouse CD45 (30-F11), APC mouse anti-mouse CD64 (X54-5/7.1), and APC-eFluor780 Armenian hamster anti-mouse FceR1a (MAR-1) were purchased from Thermo Fisher Scientific.

Flow Cytometry Processing and Staining Protocols:

For unmixing fully stained samples, a combination of single stained controls using UltraComp eBeads (Thermo Fisher Scientific) and splenocytes was used. UltraComp eBeads were stained according to the manufacturer's instructions. The spleen was collected in ice cold 2 mL Eppendorf tubes containing 500 µL of sterile PBS (-/-). The spleen was then poured into an ice cold 35 mm Petri dish containing a 0.7-µm mesh filter (Corning) and 1.5 mL of PBS (-/-) where it was mechanically disrupted to a single cell suspension using the plunger end of a 3 mL disposable syringe. Cell suspensions were spun down at 300xg for 5 min at 4 °C, after which the supernatant was removed, and the pellet was mixed/incubated with 1 mL of ACK Lysis Buffer (Gibco) for 5 min at room temperature. Lysis buffer was diluted with 9 mL of PBS (-/-), and the solution was spun down at 300xg for another 5 min at 4 °C, after which the supernatant was removed, and the pellet was resuspended in 1 mL of ice cold FACS buffer for hemocytometer cell counting and antibody staining.

Tumors were collected in ice cold 2 mL Eppendorf tubes containing 1 mL DMEM. For mechanical disruption, tumors were finely minced in their collection tubes, on ice, using dissection scissors. Following this, the suspension was prepared for enzymatic digestion by adding 40 µL of Liberase DL (28 U/mL stock), 80 µL of Liberase TL (14 U/mL stock), 40 µL of DNase I (15 mg/mL stock) [6].The final volume was brought to 2 mL with extra DMEM. The solution was then incubated at 37 °C under continuous rotation for 30 minutes. After incubation, the digested tumor solution was triturated, passed through a 70 µm cell strainer, and washed with several mL of DMEM + 10% FBS while on ice. The solution was spun at 300xg for 5 min at 4 °C and resuspended in 1 mL of fresh DMEM + 10% FBS on ice for subsequent hemocytometer cell counting and antibody staining. Fully stained samples, or compensation controls, were stained using the Live/Dead Fixable Blue Dead Cell Stain Kit (Thermo Fisher Scientific) according to the manufacturer's instructions. After, cells were washed, pelleted, and incubated with anti-CD16/CD32 for 30 min in the dark at 4 °C to block nonspecific antibody binding. Finally, blocked cells were stained with combinations of fluorochrome-conjugated antibodies for 30 min in the dark at 4 °C. Cells were fixed in 4% paraformaldehyde in PBS (-/-) for 30min at room temperature and resuspended in

fresh FACS buffer prior to acquisition. Stained cells were analyzed within 24 hours on a Cytek Aurora spectral flow cytometer. All datasets were analyzed using FlowJo software v10.7.1 (TreeStar).

FACS Protocol for scRNA-seq:

Continuing from the tumor digestion steps described in the “Flow Cytometry Processing and Staining Protocols” section, tumor suspensions were first spun down at 300xg for 5 min at 4 °C and then resuspended in Fixable Viability Stain 700 (BD Biosciences) (1:4500 dilution of FVS700 in PBS (-/-)) for 20 minutes at 4 °C. Cells were then washed with FACS buffer, spun as described before, and resuspended in 50uL of anti-CD16/32 Fc blocking antibodies in FACS buffer (1:100 dilution) for 20 min at 4 °C. After this incubation, 50uL of anti-CD19-BUV737 and anti-CD45-Pacific Blue was added (1:160 and 1:400 final dilution respectively) to the solution and allowed to incubate with tumor cells for another 30 min at 4 °C. CD19 was included to make sure tumor suspensions were not contaminated with lymph nodes, as we have found that more aggressive models can engulf tumor draining lymph nodes, leading to ~50% of recovered immune cells as B cells. Following this, cells were immediately washed with an excess of FACS buffer, spun down at 300xg for 5 min at 4 °C, and resuspended in 300 uL of FACS buffer. Suspensions were immediately taken to Stanford’s FACS facility, in which they were sorted for live, $CD45^+$ cells on a 5 laser Aria Fusion cytometer using a 130 μm nozzle. We sorted ~50,000 cells for each tumor type into DMEM + 10% FBS and adjusted the final concentration to ~1,000 cells/μl. These cells were then submitted to the Stanford Functional Genomics Facility for 10x Genomics 3’V3 library preparation and single-cell RNA sequencing.

References Cited

1 Fite, B. Z. *et al.* Immune modulation resulting from MR-guided high intensity focused ultrasound in a model of murine breast cancer. *Sci Rep* **11**, 927, doi:10.1038/s41598-020-80135-1 (2021).

2 Wu, T. *et al.* clusterProfiler 4.0: A universal enrichment tool for interpreting omics data. *The Innovation* **2**, 100141 (2021).

3 Blighe, K., Rana, S. & Lewis, M. EnhancedVolcano: Publication-ready volcano plots with enhanced colouring and labeling. *R package version* **1** (2019).

4 Kolde, R. & Kolde, M. R. Package 'pheatmap'. *R package* **1**, 790 (2015).

5 Newman, A. M. *et al.* Determining cell type abundance and expression from bulk tissues with digital cytometry. *Nat Biotechnol* **37**, 773-782, doi:10.1038/s41587-019-0114-2 (2019).

6 Cassetta, L. *et al.* 211-229 (Springer International Publishing).

Supplementary Figures and Tables

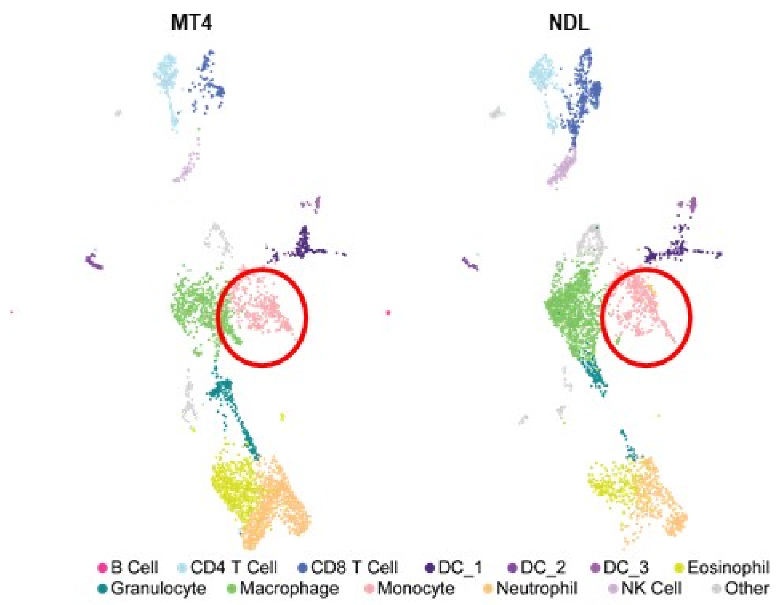

	avg_log2FC	p_val_adj		avg_log2FC	p_val_adj
Plac8	2.69365476	0	F13a1	2.79183726	0
Chil3	2.45002738	0	Mrc1	2.62538894	0
F13a1	2.30081876	0	Ccl6	2.61197882	0
Ly6c2	2.15988106	0	Ccl7	2.32385018	0
Ccl2	2.15611831	0	Ccr2	2.24743527	0
Ifitm3	2.05627428	0	S100a4	2.2203416	0
Vcan	2.01559992	0	Plac8	2.2152877	0
Ccr2	1.99333872	0	Ccl9	2.17563334	0
Ifi27l2a	1.97050046	0	Gda	2.01099054	0
Ms4a6c	1.9699529	0	Ahnak	1.9701903	0
Ccl7	1.86279937	0	Pf4	1.93263487	0
Thbs1	1.78267539	0	Lyz2	1.85293024	0
Tgfbi	1.75864018	0	Clec10a	1.81247838	0
Ms4a4c	1.74258933	0	S100a6	1.78610202	0
Lyz2	1.7193774	0	Maf	1.7760168	0
Ahnak	1.6790696	0	Pltp	1.6980119	0
Clec4a1	1.58983565	0	Emp1	1.69198005	0
Mrc1	1.52505392	0	Clec4a1	1.65427357	0
Selenop	1.50601301	0	Cd93	1.57622846	0
Ms4a6d	1.49664419	0	Pid1	1.51943944	0
Ly6e	1.43411466	0	Pira2	1.45593347	0
Ifitm6	1.42960811	0	Ccl2	1.44478281	0
Ifi204	1.41280383	0	Ifitm3	1.42427432	0
Clec4a3	1.41095571	0	Zeb2	1.41294347	0
Ms4a6b	1.40389386	0	Klf4	1.38175908	0
Pid1	1.36830633	0	Clec12a	1.38011764	0
Alox5ap	1.33235467	0	Dok2	1.37843999	0
Fcgr1	1.31027577	0	Ap2a2	1.37283985	0
Mafb	1.3081607	0	Cbr2	1.36770918	0
Wfdc17	1.29556432	0	Msr1	1.31913875	0
Slfn5	1.27387682	0	Thbs1	1.28633802	0
Klf4	1.27285851	0	Selenop	1.28276984	0
Tlr2	1.26874618	0	Fcgrt	1.28253318	0
S100a4	1.24000741	0	Rgl1	1.27226037	0
Lgmn	1.2280783	0	Cebpb	1.27104955	0
Ifi207	1.22380971	0	Jarid2	1.23220553	0
Sdc4	1.22373383	0	Pirb	1.20331232	0
Fcgr2b	1.20722334	0	Metrnl	1.19899369	0
Emp3	1.20300961	0	Cd44	1.19172827	0
Stab1	1.19892568	0	Ms4a4c	1.17705167	0
Osm	1.17850229	0	Ly6c2	1.1722814	0

Figure S1: Myeloid compartment subcluster includes Ly6c2^{hi} population. Here, we focused on the myeloid component with enhanced Ly6c2 expression, as Ly6c2 is key in aCD40 immunotherapy. In myeloid compartment subclusters, the differentially expressed genes indicate subpopulations with high Ly6c2, Ccl7 and Mrc1 expression. We used SingleR to annotate clusters based on Pearson similarity compared to purified cell bulk-RNA sequencing gene profiles to gain an overall picture of possible cell types. We then used flow cytometry markers and canonical gene markers to further annotate these cell clusters. B cells (dark pink) were CD19^{+}, CD79^{+} and Ly6d^{+}. T cells were CD3e^{+}, with CD8^{+} (dark blue) and CD4^{+} (light blue) T cells defined by CD8a and CD4, respectively. NK cells (lavender) were Klrb1b^{+} and Klrb1c^{+} (NK1.1^{+}). Eosinophils (yellow) were Siglec-F^{+}. Neutrophils (light orange) were Ly6g^{+}. Monocytes (light pink) were Ly6c^{+}, Ccr2^{+}, Mrc1^{+},

and Ccl9^{+}. Macrophages (green) were Itgam^{+} and Adgre1^{+}. DC_1 (dark purple) were Clec4a1^{+} and Cd209d^{+}. DC_2 (medium purple) were Il12b^{+}, Ccl5^{+}, Ccl22^{+}, H2-M2^{+}, H2-D1^{+}, and Siglec-H-. DC_3 (light purple) were Clec9a^{+}, Xcr1^{+} and Ppt1^{+}. Granulocytes (turquoise) were Tmem189^{+}, Sap30^{+}, and Idha^{+}. In each model, the top 40 genes within this cluster are listed together with the average $\log_2$ fold change (FC) and adjusted P value compared with cells outside of this cluster.

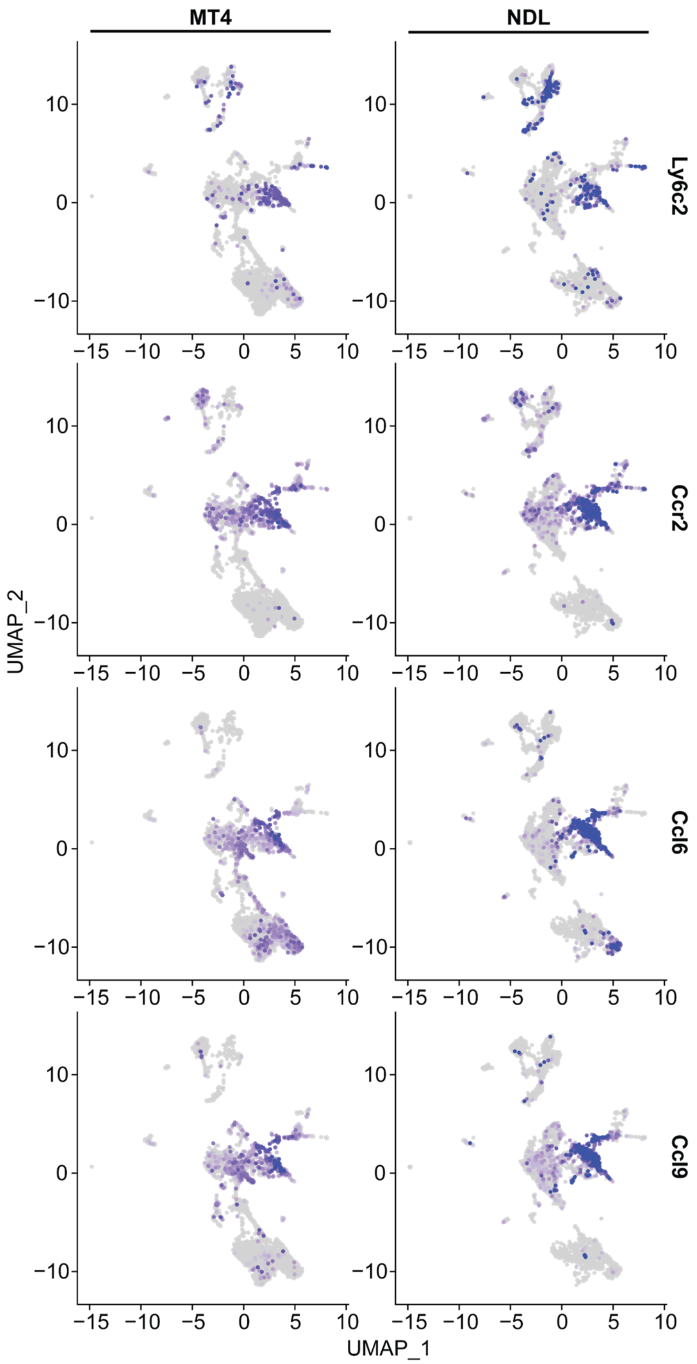

Figure S2. Myeloid compartment subpopulation of single-cell sequencing data acquired from treatment-naïve MT4 (pancreatic) and NDL (breast) cancer models, as characterized by monocyte markers. The myeloid compartment subpopulation (cluster 4) is rich in expression of Ly6c2, Ccr2, Ccl6, and Ccl9 in both the MT4 and NDL TMEs.

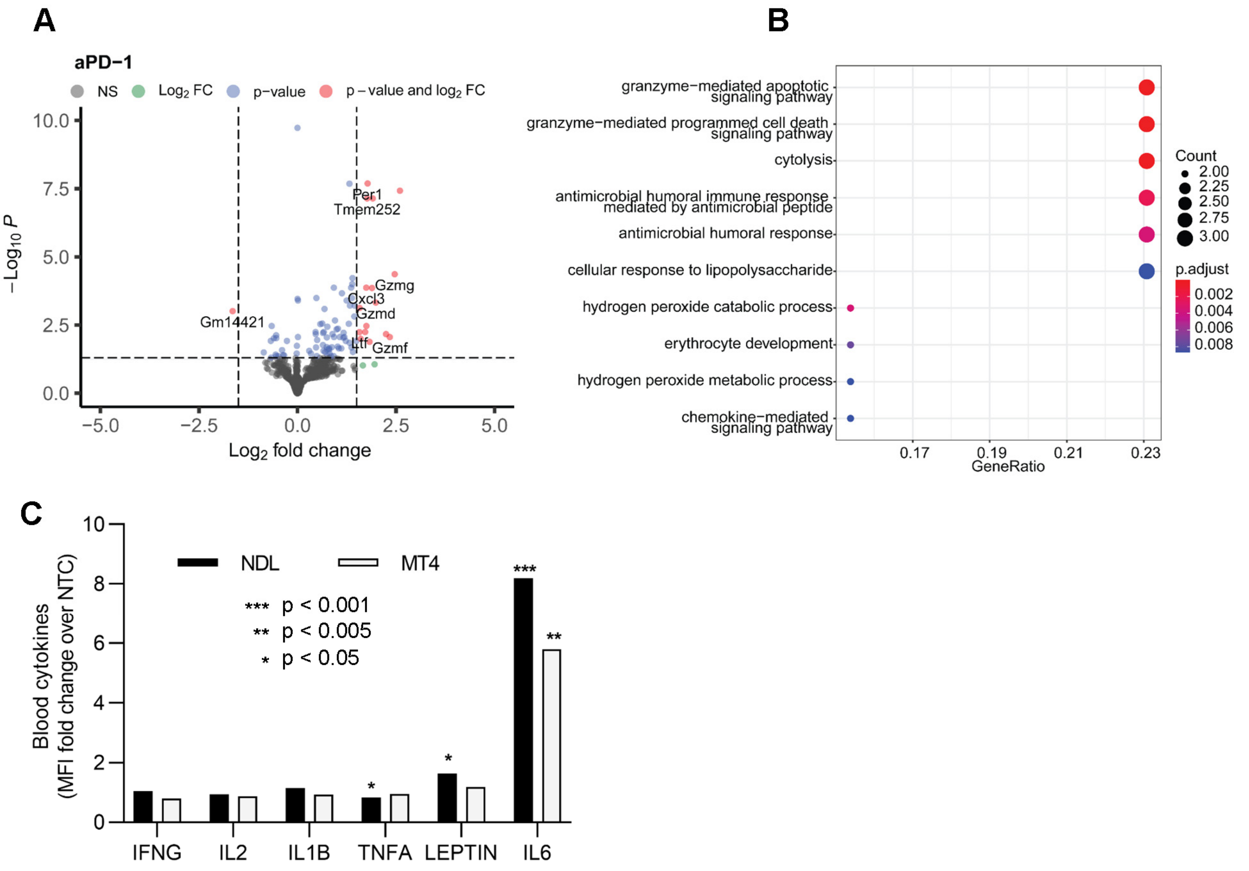

Figure S3. **Impacts of single treatments in the MT4 model.** A-B) In the MT4 model (as previously observed in the NDL model), treatment with aPD-1 alone has a limited impact on immune-related gene expression. Based on the protocol shown in Fig. 3A, bulk RNA sequencing was performed on tumor tissue at 72 hours after a single treatment with aPD-1. A) Volcano plot showing aPD-1 treatment modulates 52 genes (adjusted p value < 0.05 and a $\log_2$ fold change > 2), including three granzyme-family genes, Cxcl3, Il6, CTLA4 and MMP8, which are all enhanced between 2 and 6 fold. B) Gene ontology enrichment analysis revealed aPD-1 treatment does not enrich immune-related or toll-like receptor (TLR) processes and does not modulate MT4 cancer gene expression; the granzyme-mediated apoptotic signaling pathway is upregulated, however. C) For ablation alone, blood cytokine average fold change levels of NDL and MT4 tumors (n=5) five hours compared to no treatment control (NTC) mice. Five hours after ablation, blood was collected for multiplexed Luminex analysis. The mean fluorescence intensity (MFI) over NTC is the MFI fold change of treated tumors when compared to NTC. Asterisks denote significance based on a nonparametric t-test of the specific cytokine after ablation as compared with before ablation.

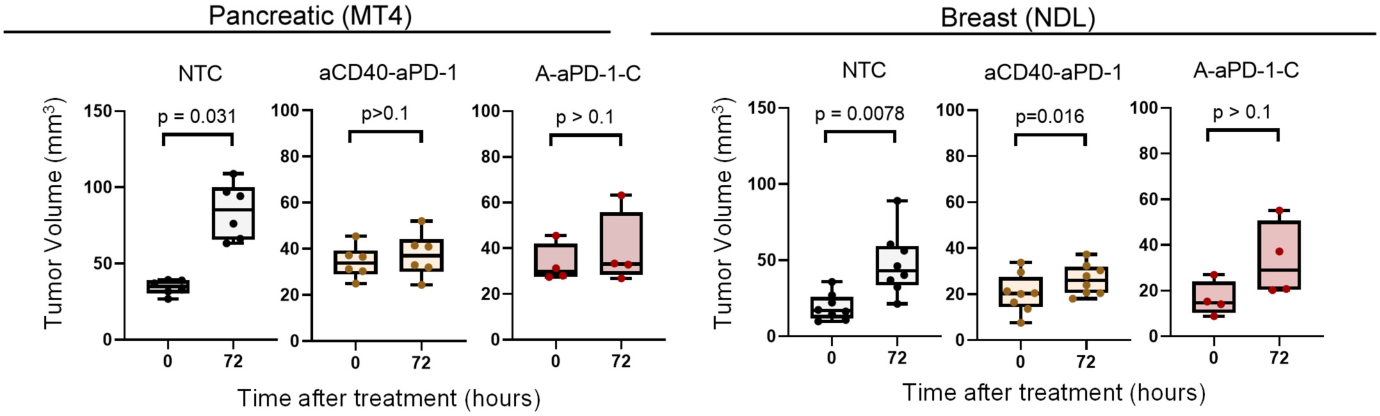

Figure S4. 3-Day tumor growth in 2-component treatments. Growth of tumors between start of treatment and tumor sequencing 72 hours later. We consider only the distant tumors in the ablated cohort as the treated tumor cannot always be accurately assessed immediately after treatment. A-aPD-1-C: contralateral tumor in mice receiving ablation + aPD-1. aCD40-aPD-1: mice receiving CD40 + aPD-1. NTC: no treatment control cohort.

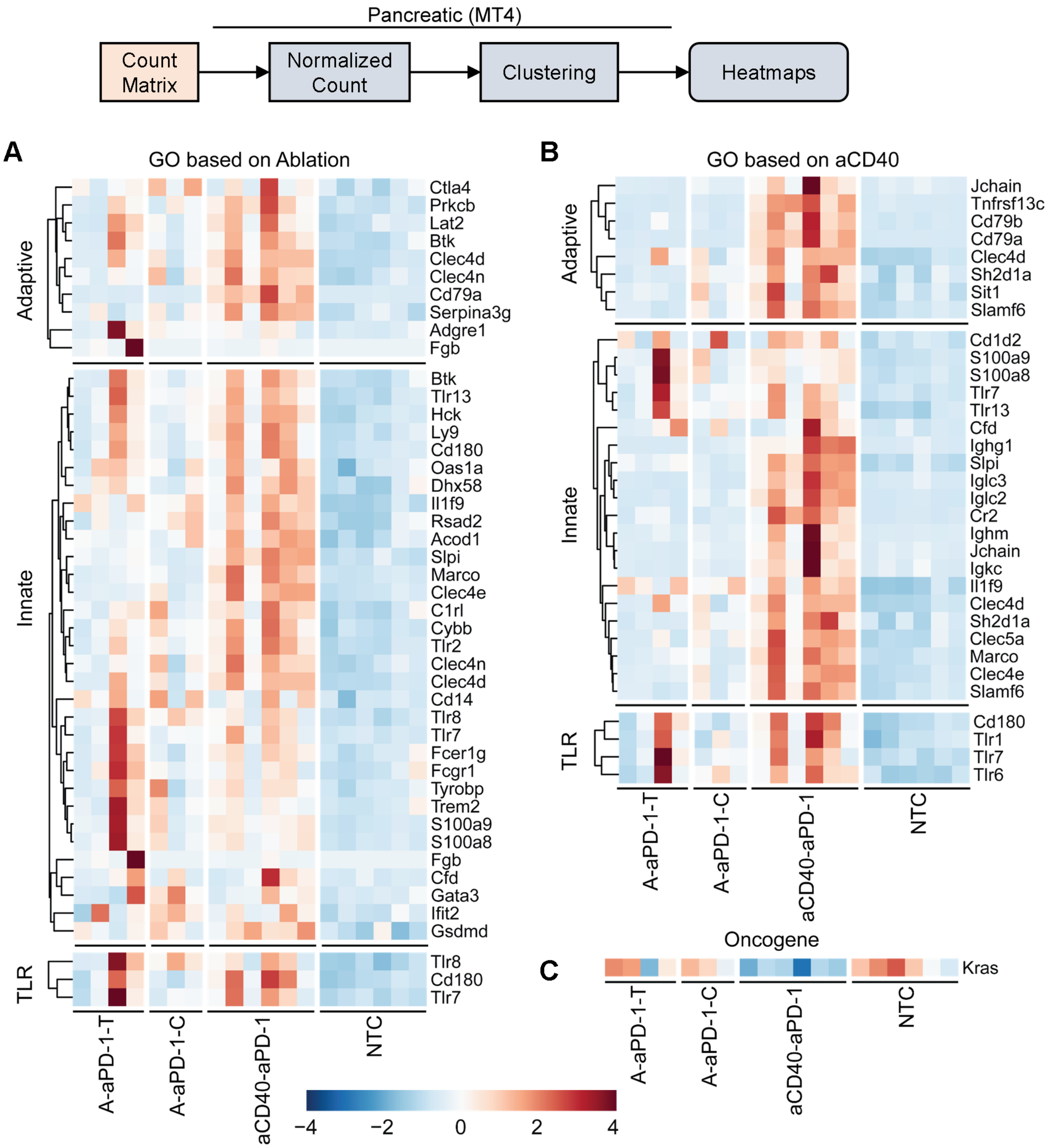

Figure S5. In the MT4 model, ablation + aPD-1 and CD40 + aPD-1 combination treatments induce responses in key immune pathways. Based on the treatment protocol in Fig. 3A, bulk RNA sequencing data were analyzed to assess immune-related and cancer-related gene ontologies. gene ontology analysis based on differentially expressed genes according to treatment by ablation + aPD-1 (A) or aCD40 + aPD-1 (B) highlighted upregulated genes in key immune pathways including the adaptive immune (GO:0002819), innate immune (GO:0045088), and toll-like receptor (TLR) (GO:0002224) ontologies. Enrichments were more prominent with aCD40 and aPD-1 combination therapy than with the ablation and aPD-1 therapy. C) Both therapies modulated cancer-associated gene Kras. A-aPD-1-T: directly-treated tumor in mice receiving ablation + aPD-1. A-aPD-1-C: contralateral tumor in mice receiving ablation + aPD-1. CD40-aPD-1: mice receiving CD40 + aPD-1. NTC: no treatment control mice. Gene ontology analysis was performed on genes differentially expressed with a $\log_2$ fold-change of 1.5 and an adjusted p value less than 0.05.

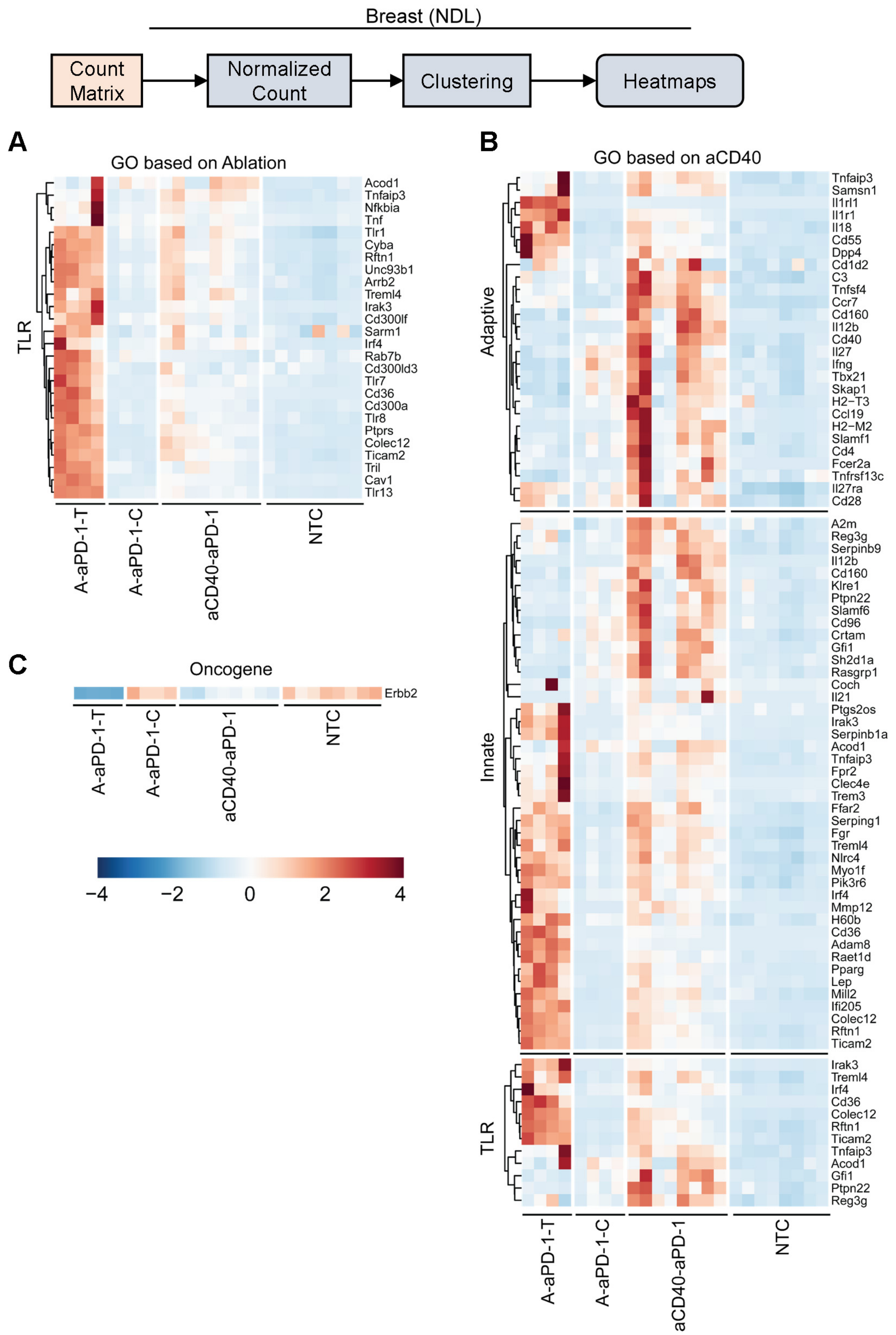

Figure S6. In the NDL model, ablation + aPD-1 and aCD40 + aPD-1 combination treatments induce responses in key immune and TLR pathways. Heatmap of key enriched gene ontology pathways. Based on the treatment protocol in Fig. 3A, bulk RNA sequencing data were analyzed to assess immune-related and cancer-related gene ontologies. A) Gene ontology analysis based on differentially expressed genes resulting from treatment with ablation + aPD-1 highlighted upregulated genes in the toll-like receptor (TLR) pathway (GO:0002224). In contrast, Gene ontology analysis based on treatment with aCD40 + aPD-1 (B) demonstrated upregulated genes in immune pathways including the adaptive immune (GO:0002819) and innate immune (GO:0045088) ontologies. C) Downregulation of key cancer gene Erbb2 was greater in the treated than in the distant tumor as a result of ablation. A-aPD-1-T: directly-treated tumor in mice receiving ablation + aPD-1. A-aPD-1-C: contralateral tumor in mice receiving ablation + aPD-1. CD40-aPD-1: mice receiving CD40 + aPD-1. NTC: no treatment control mice. Gene ontology analysis was performed on genes differentially expressed with a $\log_2$ fold-change of 1.5 and an adjusted p value of less than 0.05.

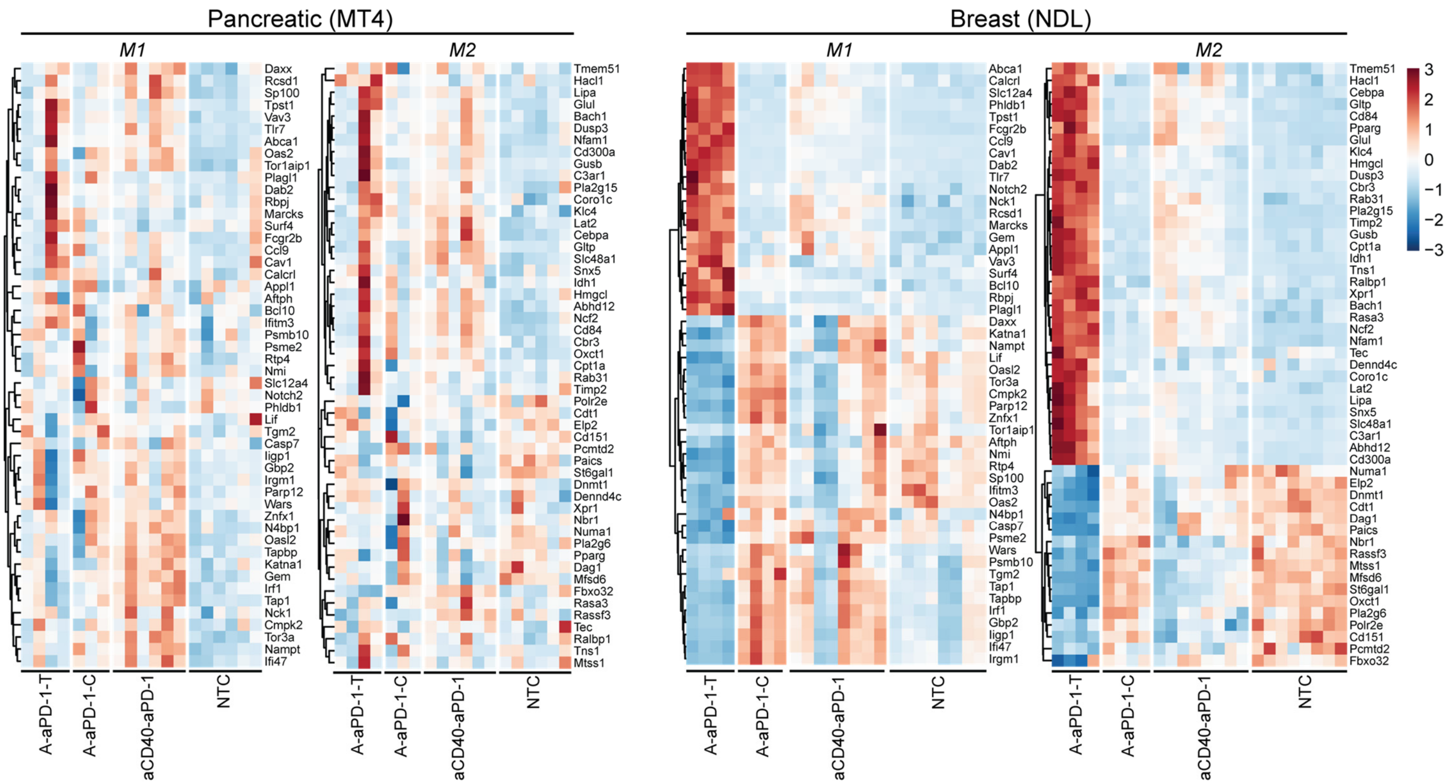

Figure S7: MRgFUS ablation modulates macrophage phenotype in the inflamed NDL tumor phenotype. Heatmap of key enriched gene ontology pathways. Bulk RNA sequencing data acquired under the treatment protocol in Fig. 3A were analyzed to determine the macrophage phenotype resulting from each treatment using the M1 and M2 macrophage phenotypes as defined in [36]. In the MT4 pancreatic tumor model (left panel), the directly-ablated tumor macrophage phenotype was not consistently altered. In the NDL breast cancer model (right panel), macrophage phenotype was consistently modulated with both increases and decreases in subsets of both M1 and M2 phenotypes in the directly-ablated tumors. A-aPD-1-T: directly-treated tumor in mice receiving ablation + aPD-1. A-aPD-1-C: contralateral tumor in mice receiving ablation + aPD-1. CD40-aPD-1: mice receiving CD40 + aPD-1. NTC: no treatment control mice.

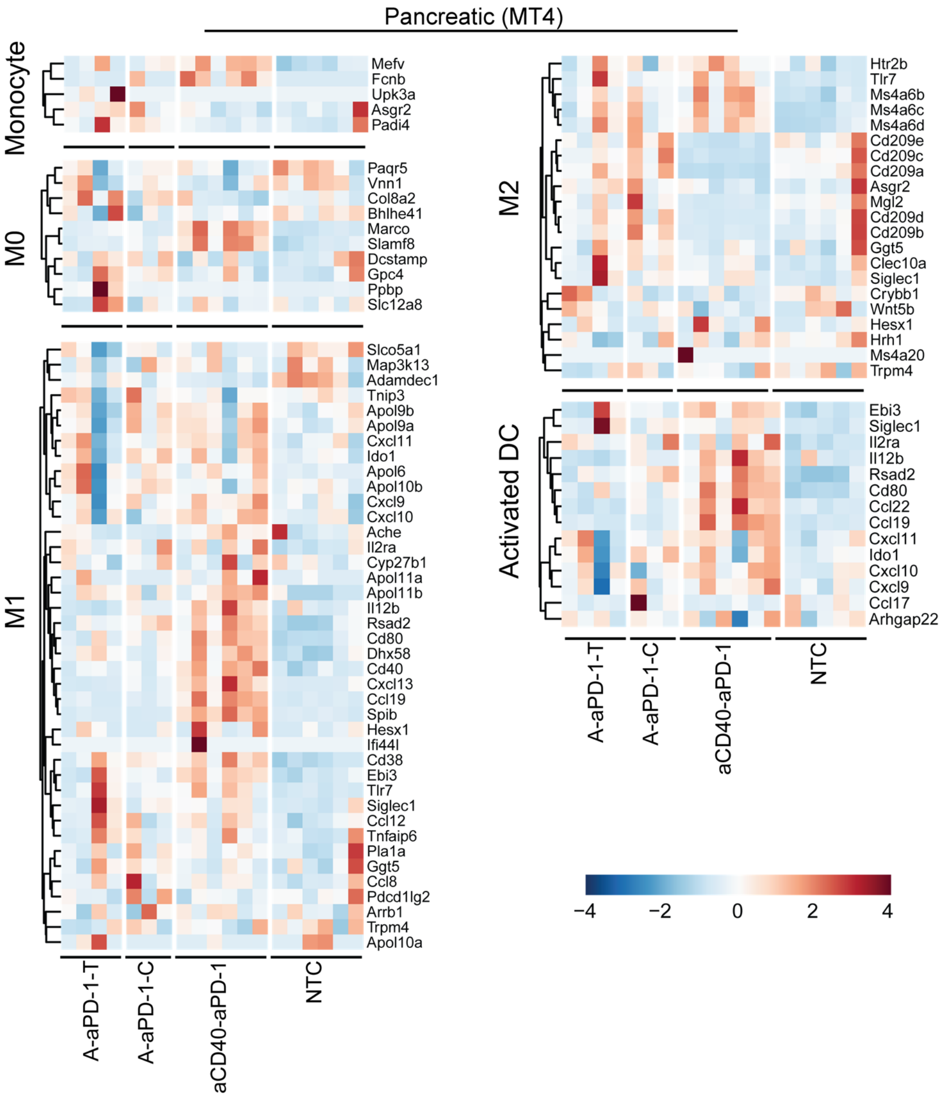

Figure S8: Effect of ablation + aPD-1 and aCD40 + aPD-1 combination treatments on macrophage and monocyte phenotypes in the MT4 pancreatic cancer model. Bulk RNA sequencing data acquired under the treatment protocol in Fig. 3A were analyzed using CIBERSORTx based on the LM22M deconvolution signature matrix, and hierarchical heatmap clustering of myeloid subsets (monocyte, M0, M1, M2, and activated dendritic cells (DCs)). aCD40-aPD-1 combination treatment upregulated key genes in M1 macrophages and activated DCs. aCD40 + aPD-1 treatment altered genes related to the M1 phenotype and DC activation to a greater extent than genes in the other myeloid compartments. Ablation-based treatments did not consistently alter macrophage (M1, M2 or resting (M0)) and monocyte phenotypes or DC activation. A-aPD-1-T: directly-treated tumor in mice receiving ablation + aPD-1. A-aPD-1-C: contralateral tumor in mice receiving ablation + aPD-1. CD40-aPD-1: mice receiving CD40 + aPD-1. NTC: no treatment control mice.

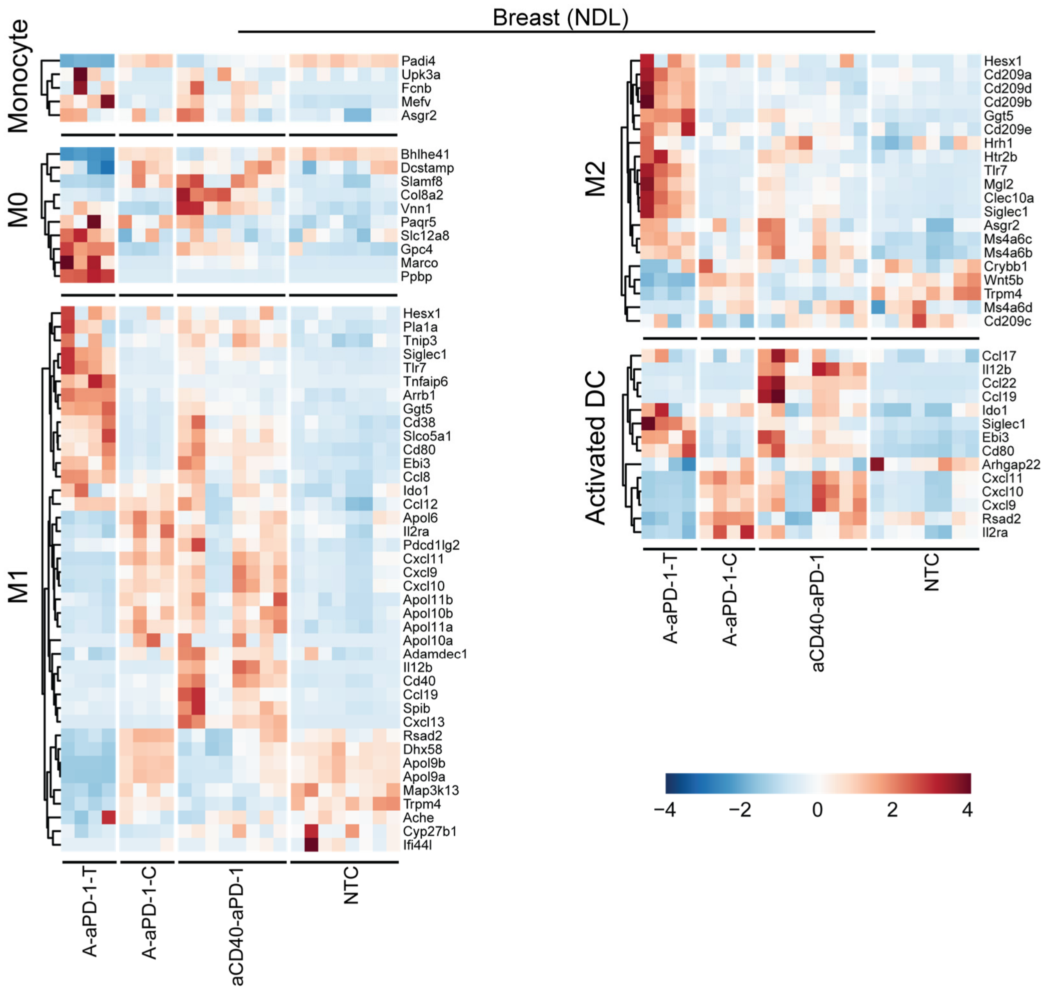

Figure S9. Effect of ablation + aPD-1 and aCD40 + aPD-1 combination treatments on macrophage and monocyte phenotypes in the NDL breast cancer model. Bulk RNA sequencing data acquired under the treatment protocol in Fig. 3A were analyzed using CIBERSORTx based on the LM22M deconvolution signature matrix, and hierarchical heatmap clustering of myeloid subsets (monocyte, M0, M1, M2, and activated dendritic cells (DCs)). Ablation + aPD-1 treatment altered components of the M0 (resting macrophage), M1 and M2 macrophage phenotypes with a greater Z score compared to aCD40 + aPD-1 treatment, and these components were largely complementary to those modified by aCD40. Treatment with aCD40 + aPD-1 altered DC activation more prominently than ablation + aPD-1. A-aPD-1-T: directly-treated tumor in mice receiving ablation + aPD-1. A-aPD-1-C: contralateral tumor in mice receiving ablation + aPD-1. CD40-aPD-1: mice receiving CD40 + aPD-1. NTC: no treatment control mice.

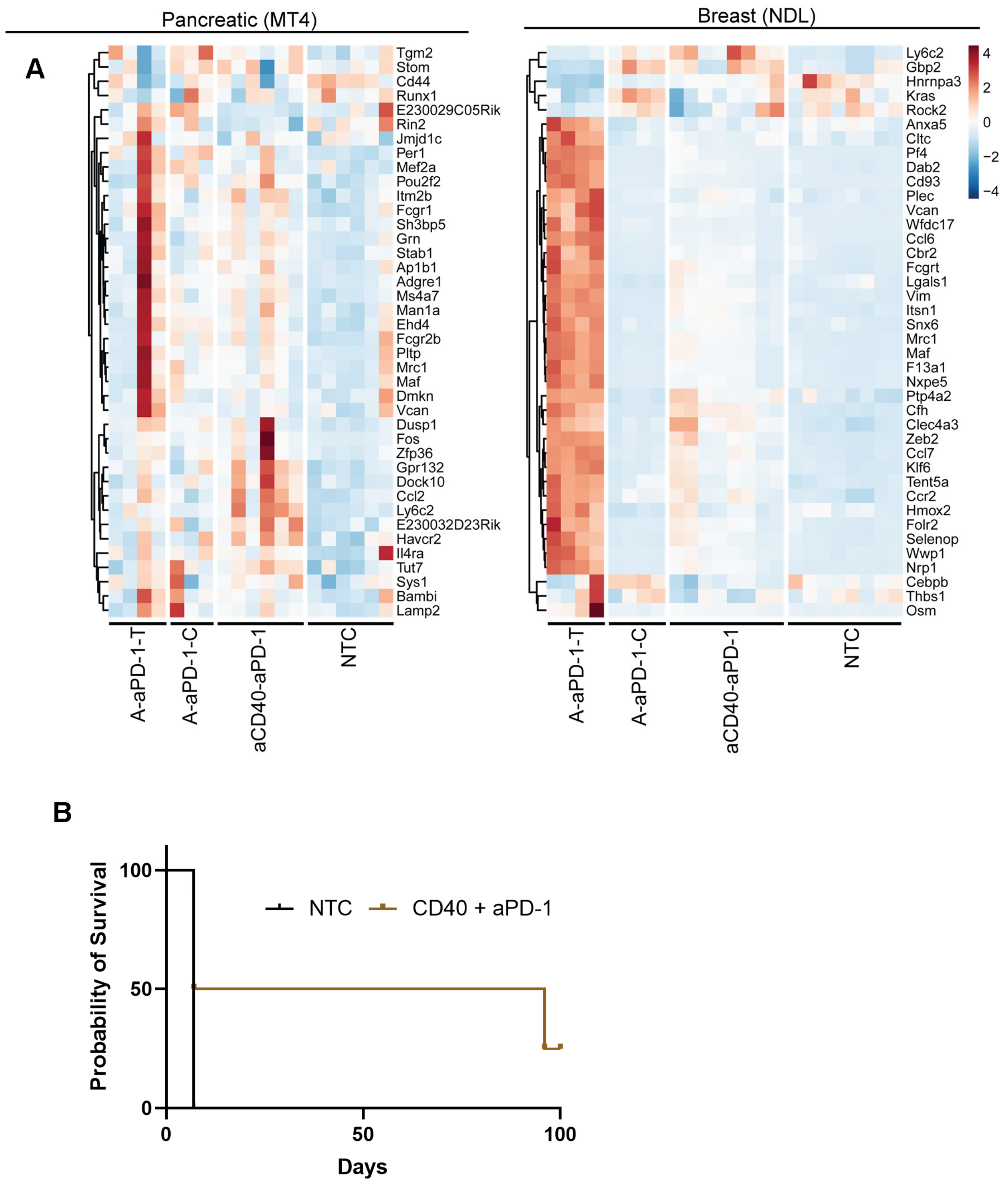

Figure S10. Further effects of immunotherapy. A) Effect of ablation + aPD-1 and aCD40 + aPD-1 combination treatments on the monocyte compartment subpopulation as defined in Fig. 2 in the MT4 (pancreatic) and NDL (breast) cancer models. Heatmap of monocyte gene set derived from single cell RNA sequencing UMAP clustering in MT4 pancreatic and NDL breast tumor models. Ablation + aPD-1 did not significantly alter monocyte subpopulation phenotypes in the MT4 pancreatic cancer model (left panel), but significantly upregulated many of the Ccl and Clec family genes in the NDL breast cancer model (right panel). In contrast, aCD40 + aPD-1 upregulated Ly6c2 in both tumor models. B) Survival of NDL mice treated with aCD40+ aPD-1 as compared with the NTC model. In the NDL model, survival was extended based on the two-component treatment. Both models were treated based on the protocol in Fig. 3A, and bulk RNA sequencing data were analyzed. A-aPD-1-T: directly-treated tumor in mice receiving ablation + aPD-1. A-aPD-1-C: contralateral tumor in mice receiving ablation + aPD-1. CD40-aPD-1: mice receiving CD40 + aPD-1. NTC: no treatment control mice.

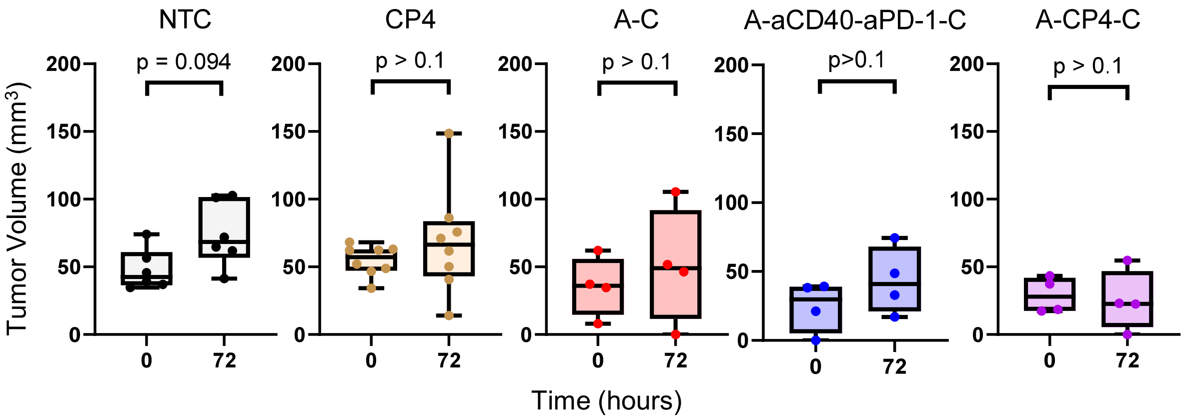

Figure S11. Three-day MT4 tumor growth in multi-component ablation-immunotherapy treatments. Growth of tumors between start of treatment and tumor sequencing 72 hours later. We consider only the distant tumors (-C) in the ablated cohorts as the treated tumor cannot always be accurately assessed immediately after treatment. CP4: mice receiving aCD40 + aPD-1 + aCTLA-4; A-C: mice receiving ablation; A-aCD40-aPD-1-C: mice receiving ablation + aCD40 + aPD-1; A-CP4-C: mice receiving ablation + aCD40 + aPD-1 + aCTLA-4; NTC: no treatment control cohort.

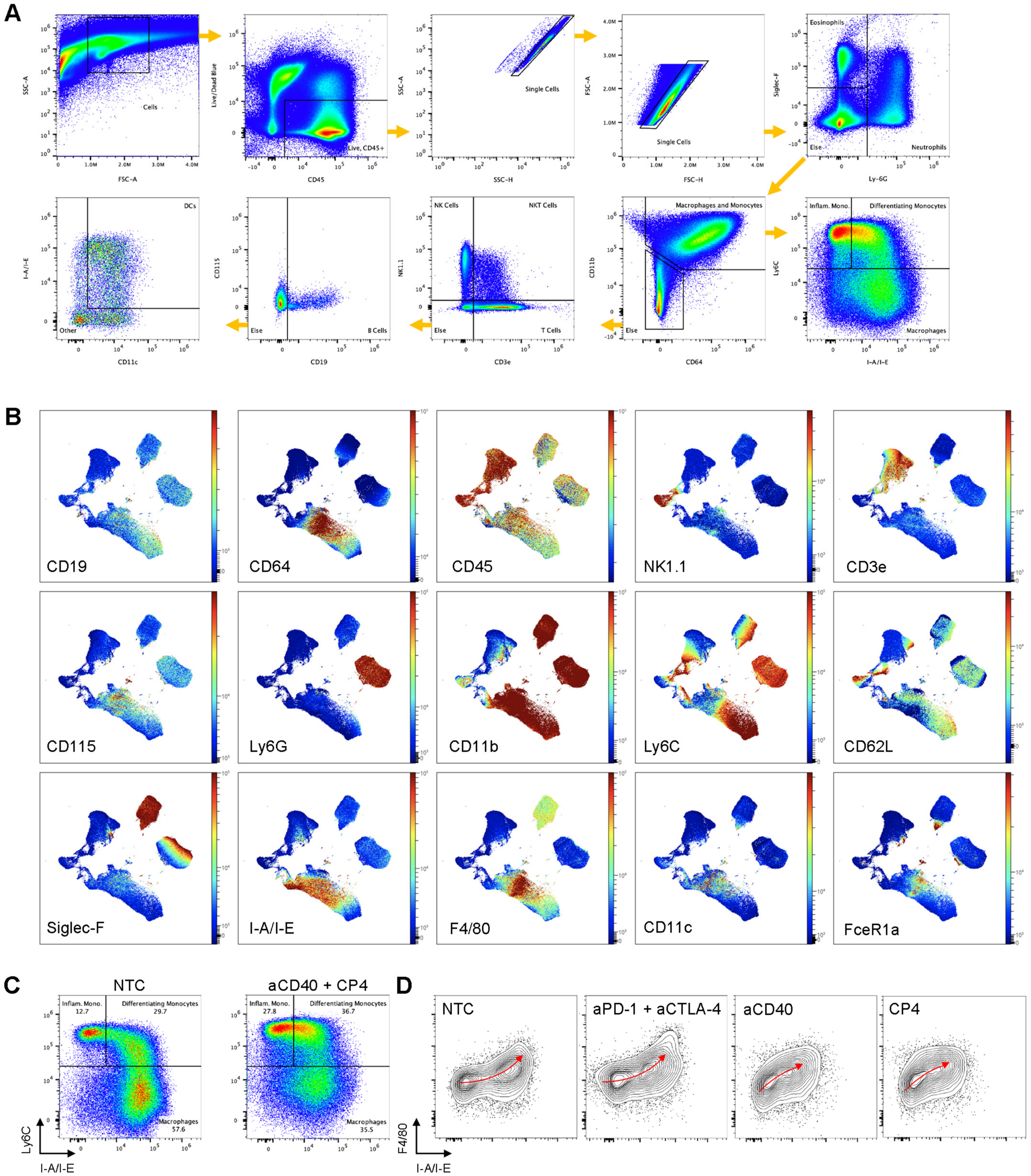

Figure S12. Spectral cytometry overview in MT4 tumors. MT4 tumor-bearing mice were treated based on the protocol in Fig. 3A, comparing a one-time injection of aCD40 to an injection of aCD40 combined with the two checkpoint inhibitors, aPD-1 and aCTLA-4 (denoted CP4), or aPD-1 and aCTLA-4 alone, using spectral cytometry at 72 hrs. A) Representative gating strategy used to manually identify cell types. Yellow arrows represent sequential gating steps. Dot plots shown were used from a concatenated file containing all NTC, aCD40, and CP4 events. The following combinations of surface makers were used to label subsets within single, live, CD45$^+$ cells: eosinophils were Siglec-F$^+$, neutrophils were Ly6G$^+$, monocytes were CD11b$^+$CD64$^+$Ly-6C^{hi} (inflammatory monocytes were I-A/I-E- and differentiating monocytes were I-A/I-E$^+$), macrophages were CD11b$^+$CD64$^+$Ly6C^{lo}, T cells were CD3e$^+$NK1.1$^-$, NK cells were CD3e$^-$NK1.1$^+$, NKT cells were NK1.1$^+$CD3e$^+$, B cells were CD19$^+$, and dendritic cells (DCs) were CD64$^-$CD11c$^+$I-A/I-E$^+$. B) Multigraph color mappings of

15 fluorescent parameters (mapped on NTC UMAP containing 250,000 events across 5 replicates). Color bars represent each marker expression intensity, such that red is high expression and blue is low to zero. C) Pseudocolor dot plots of CD11b⁺CD64⁺ monocytes and macrophages from NTC and aCD40 + CP4 treatments (128226 events each). D) Contour plots of CD11b⁺CD64⁺Ly6C⁺ monocytes from NTC, aPD-1 + aCTLA-4, aCD40, and CP4 treatments. Red arrows represent a hypothesized differentiation trajectory for inflammatory monocytes.

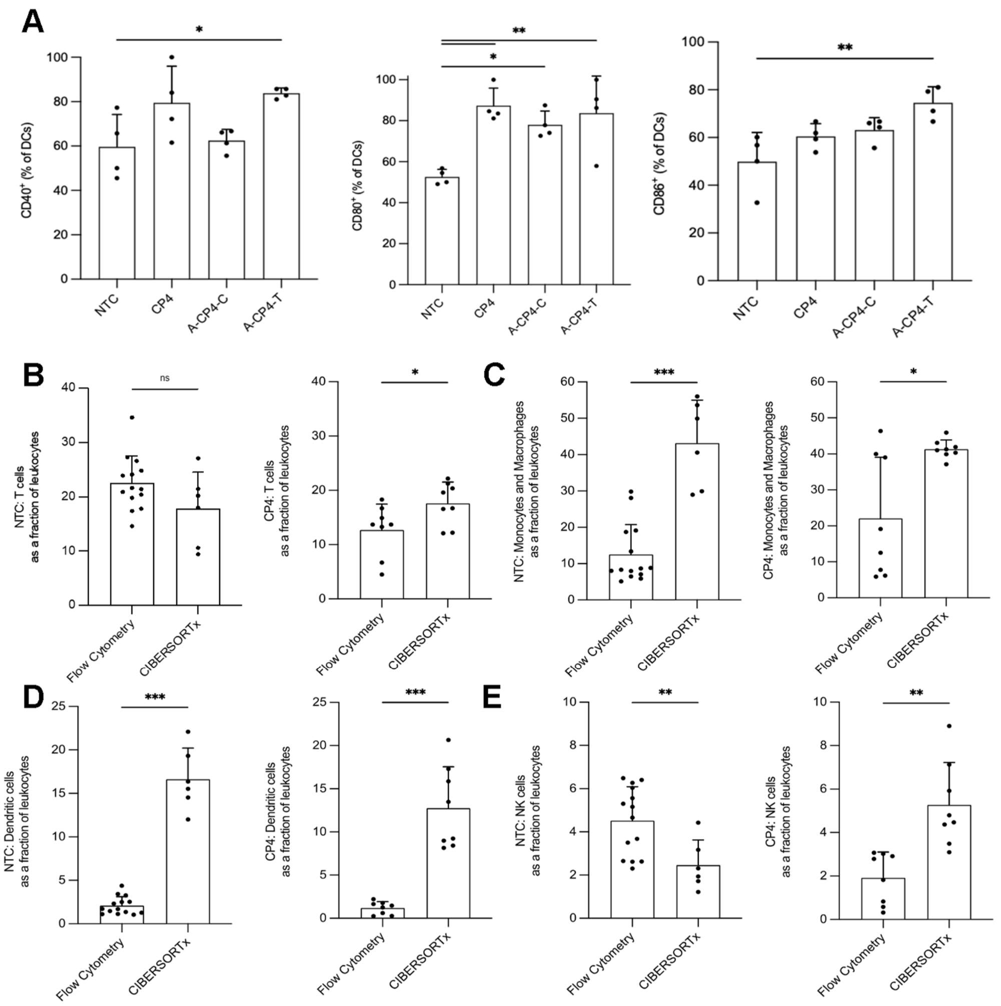

Figure S13: **Results for spectral flow cytometry and CIBERSORTx in the MT4 pancreatic cancer model.** A) Spectral cytometry results for activated dendritic cells as a function of treatment. CP4: mice receiving aCD40 + aPD-1 + aCTLA-4; A-CP4-C: contralateral tumor in mice receiving ablation + aCD40 + aPD-1 + aCTLA-4; A-CP4-T: treated tumor in mice receiving ablation + aCD40 + aPD-1 + aCTLA-4. B-E) Comparison of major cell populations estimated by spectral flow cytometry after gating on live, single, CD45⁺ immune cells versus results obtained by bulk sequencing without sorting followed by CIBERSORT analysis in no treatment control (NDL) and CP4 cohorts. B) T cells, C) dendritic cells, D) macrophages and monocytes, E) NK cells. Statistical analyses were performed using one-way ANOVA with Tukey's multiple comparisons test. ns = non-significant, * = $p < 0.05$, ** = $p < 0.01$, *** = $p < 0.001$, **** = $p < 0.0001$.

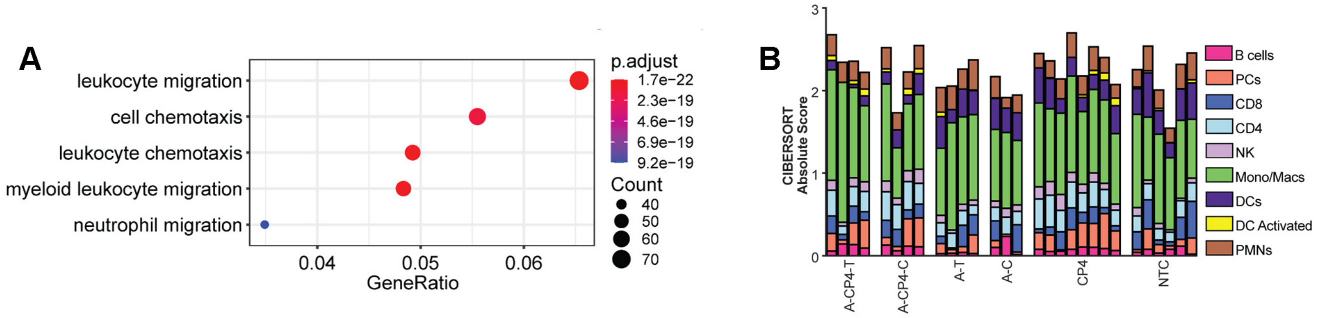

Figure S14: **In the MT4 pancreatic tumor model, summary of the bulk sequencing and CIBERSORT absolute score summary for data presented in Fig. 6.** A) Genes altered by ablation + CP4 treatment in the directly-treated tumor were enriched in leukocyte migration, chemotaxis processes, myeloid leukocyte and neutrophil migrations. B) CIBERSORT absolute score. Activated dendritic cells increased in both treated (A-CP4-T) and distant (A-CP4-C) tumors as a result of ablation + CP4 treatment compared to ablation alone in either the treated (A-T) or distant sites (A-C) and the no treatment control (NTC) cohort. This result was comparable to CP4 systemic treatment.

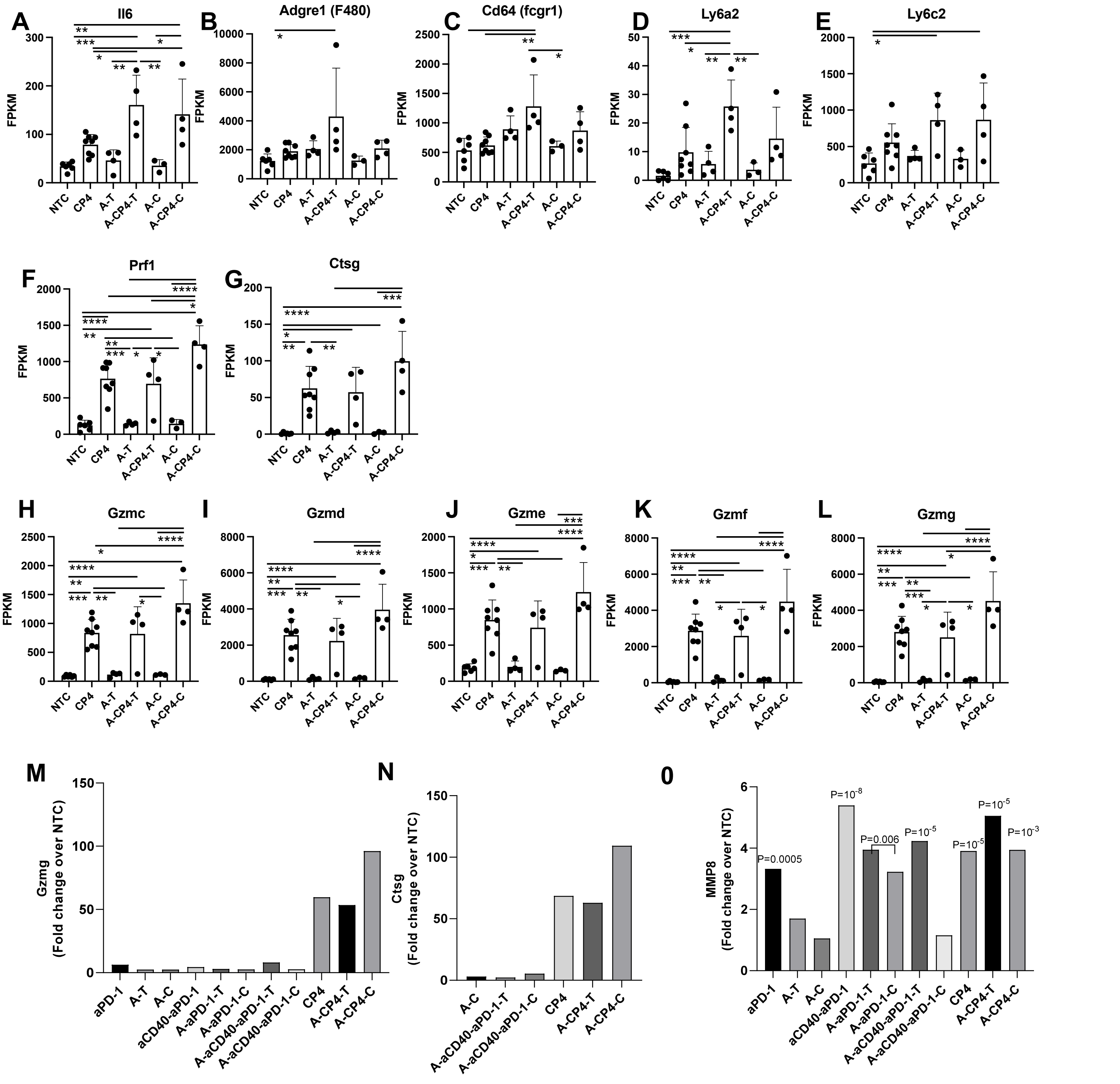

Figure S15. CP4 treatment increases Prf1 and broadly increases granzyme expression, whereas CP4 in combination with ablation further enhances monocyte and macrophage markers in the MT4 pancreatic cancer model. Bulk sequencing data resulting from the treatment of the MT4 tumor model using the protocol in Fig. 3A or 6A were analyzed. CP4 or CP4 + ablation altered markers of myeloid biology. The combination of ablation and CP4 upregulated A) the inflammatory cytokine Il6, B) the macrophage marker F4/80 as well as C) Cd64, D) Ly6a2 and E) Ly6c2. CP4 treatment and CP4 with ablation upregulated F) Prf1, G) Ctsg, (H) Gzmc, (I) Gzmd, (J) Gzme, (K) Gzmf, and (L) Gzmg. Fold change for M) Gsmg, N) Ctsg for treatments that exhibited significantly increased fold changes (adjusted P value <0.05) compared to the no treatment control (NTC) cohort. O) Fold change for MMP8 compared with NTC with associated P Values. A-CP4-T: directly-treated tumor in mice receiving ablation + CP4 (aCD40 + aPD-1 + aCTLA-4) treatment. A-CP4-C: contralateral tumor in mice receiving ablation + CP4. A-aCD40-aPD-1-T: directly-treated tumor in mice receiving ablation + aCD40 + aPD-1. A-aCD40-aPD-1-C: contralateral tumor in mice receiving ablation + aCD40 + aPD-1. A-aPD-1-T: directly-treated tumor in mice receiving ablation + aPD-1. A-aPD-1-C: contralateral tumor in mice receiving ablation + aPD-1. A-T: directly-

treated tumor in mice receiving ablation only. A-C: contralateral tumor in mice receiving ablation only. NTC: no treatment control mice. For graphs A-L, data are presented as mean ± SD. Statistical analyses were performed using one-way ANOVA with Tukey's multiple comparisons test. * = $p < 0.05$, ** = $p < 0.01$, *** = $p < 0.001$, **** = $p < 0.0001$.

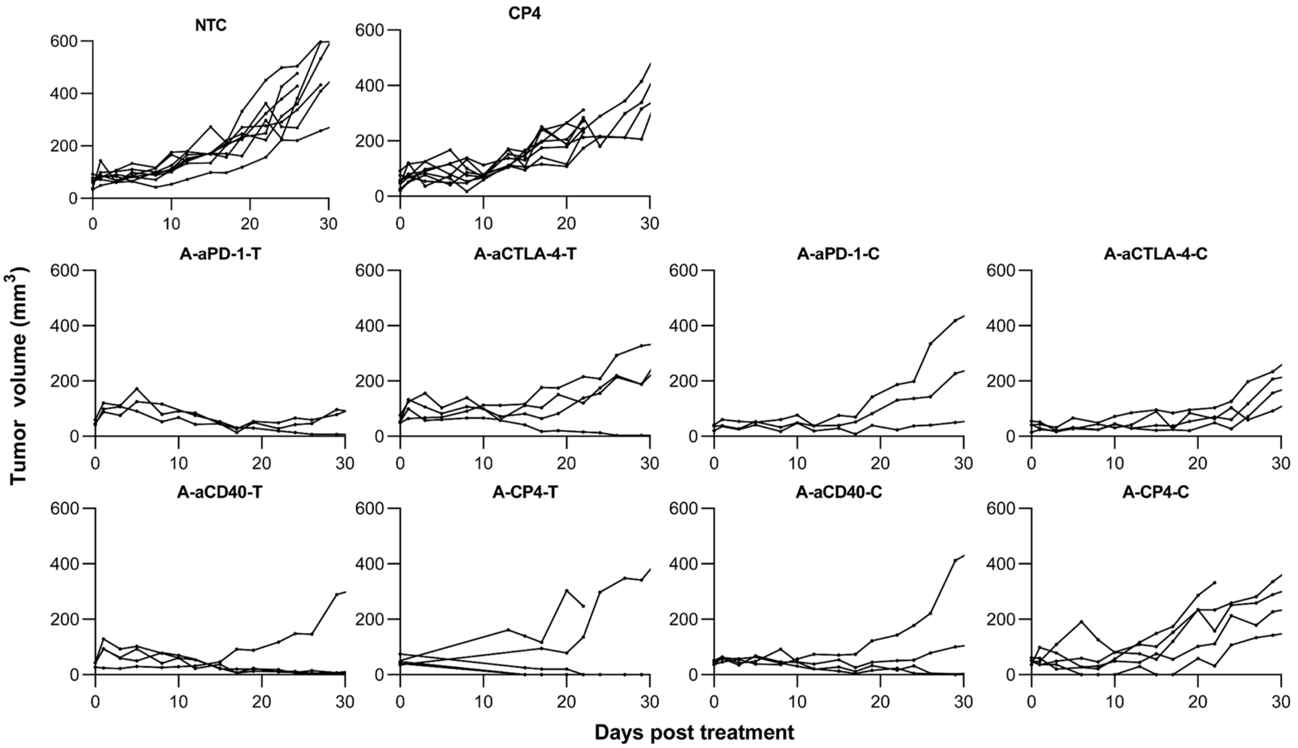

Figure S16. Tumor volume results for ablation-immunotherapy treatments reported in Fig. 7 and additional controls.

Abbreviations:
NTC: no treatment control.
CP4: mice receiving aCD40 + aPD-1 + aCTLA-4.
A-aPD-1-T: treated tumor for mice receiving ablation + aPD-1.
A-aPD-1-C: contralateral tumor for mice receiving ablation + aPD-1.
A-aCTLA-4-T: treated tumor for mice receiving ablation + aCTLA-4.
A-aCTLA-4-C: contralateral tumor for mice receiving ablation + aCTLA-4.
A-aCD40-T: treated tumor for mice receiving ablation + aCD40.
A-aCD40-C: contralateral tumor for mice receiving ablation + aCD40.
A-CP4-T: treated tumor for mice receiving ablation + aCD40 + aPD-1 + aCTLA-4.
A-CP4-C: contralateral tumor for mice receiving ablation + aCD40 + aPD-1 + aCTLA-4.

Supplementary Tables

Table S1. P values for CIBERSORTx for MT4 tumors comparing ablation + aPD-1 in treated (-T) and distant (-C) tumors and the aCD40-aPD-1 combination treatment as compared to the no treatment control cohort based on the data shown in Fig. 4B-C. Comparisons based on the Mann Whitney test.

	A-aPD-1-T	A-aPD-1-C	aCD40-aPD-1
**Plasma cells**	0.914286	0.714286	0.002165
**$CD8^+$ T cells**	0.352381	>0.999999	0.132035
**Regulatory T cells**	0.357143	0.5	0.145022
**Resting NK cells**	0.8	>0.999999	0.707792
**Activated NK cells**	0.761905	0.380952	0.818182
**Resting dendritic cells**	0.914286	0.714286	0.309524
**Activated dendritic cells**	0.019048	0.547619	0.008658
**B cells**	0.257143	0.166667	0.002165
**$CD4^+$ T cells**	0.814286	0.095238	0.179654
**Monocytes and Macrophages**	0.009524	0.02381	0.004329
**PMNs**	0.761905	0.166667	0.699134

Table S2. P values for CIBERSORTx for NDL tumors comparing ablation + aPD-1 in treated (-T) and distant (-C) tumors and the aCD40-aPD-1 combination treatment as compared to the no treatment control cohort based on the data shown in Fig. 4E-F. Comparisons based on the Mann Whitney test.

	**A-aPD-1-T**	**A-aPD-1-C**	**aCD40-aPD-1**
**Plasma cells**	0.019048	0.47619	0.413586
**$CD8^+$ T cells**	0.27619	0.019048	0.059274
**Regulatory T cells**	0.4	0.133333	0.000666
**Resting NK cells**	0.033333	>0.999999	>0.999999
**Activated NK cells**	0.114286	0.009524	0.000666
**Resting dendritic cells**	0.561905	0.257143	0.001332
**Activated dendritic cells**	0.033333	>0.999999	0.000666
**B cells**	0.009524	0.019048	0.000666
**$CD4^+$ T cells**	0.066667	0.452381	0.370629
**Monocytes and Macrophages**	0.009524	0.114286	0.000666
**PMNs**	0.009524	0.009524	0.01998

Table S3. P values for CIBERSORTx for MT4 tumors comparing ablation, CP4, and ablation + CP4 in treated (-T) and distant (-C) tumors as compared to the no treatment control cohort based on the data shown in Fig. 6G-J. Comparisons based on the Mann Whitney test.

P values for CIBERSORTx

	**A-CPT-T**	**A-CP4-C**	**A-T**	**A-C**	**CP4**
**Plasma cells**	0.171429	0.352381	0.914286	0.166667	0.081252
**CD8$^+$ T cells**	0.47619	0.47619	0.47619	0.714286	0.344988
**Follicular helper T cells**	0.257143	0.352381	0.352381	0.714286	0.141858
**Regulatory T cells**	0.714286	0.857143	0.904762	>0.999999	0.445221
**Resting NK cells**	0.238095	0.32381	0.438095	0.857143	0.056943
**Activated NK cells**	0.109524	0.019048	0.72381	0.380952	0.056943
**Resting dendritic cells**	0.009524	0.038095	0.609524	0.904762	0.141858
**Activated dendritic cells**	0.185714	0.27619	0.657143	0.166667	0.768898
**B cells**	0.114286	0.171429	0.171429	0.714286	0.282384
**CD4$^+$ T cells**	0.066667	0.009524	0.761905	0.380952	0.000666
**Monocytes and Macrophages**	0.114286	0.914286	0.47619	0.714286	0.490842
**PMNs**	0.761905	0.47619	0.352381	0.261905	0.282384

Table S4. Gene ontology enrichment tables for MT4 and NDL tumor models and treatment types.

NDL

Treatment	ID	Description	GeneRatio	BgRatio	pvalue	p.adjust	FDR
A-aPD-1-T	GO:0050900	leukocyte migration	206/6133	360/23328	9.26E-36	5.81E-32	3.19E-32
	GO:0009611	response to wounding	256/6133	492/23328	1.18E-34	3.70E-31	2.03E-31
	GO:0042060	wound healing	196/6133	349/23328	1.45E-32	3.03E-29	1.66E-29
	GO:0003012	muscle system process	218/6133	408/23328	8.03E-32	1.26E-28	6.92E-29
	GO:0060326	cell chemotaxis	174/6133	303/23328	1.19E-30	1.49E-27	8.19E-28
aCD40-aPD-1	GO:0050900	leukocyte migration	186/3530	360/23328	5.86E-60	3.50E-56	2.02E-56
	GO:0007159	leukocyte cell-cell adhesion	173/3530	345/23328	2.29E-53	6.84E-50	3.94E-50
	GO:0022407	regulation of cell-cell adhesion	194/3530	427/23328	3.25E-51	6.47E-48	3.73E-48
	GO:1903037	regulation of leukocyte cell-cell adhesion	155/3530	309/23328	6.39E-48	9.56E-45	5.51E-45
	GO:0060326	cell chemotaxis	153/3530	303/23328	8.71E-48	1.04E-44	6.00E-45

MT4

Treatment	ID	Description	GeneRatio	BgRatio	pvalue	p.adjust	FDR
A-T	GO:0001991	regulation of systemic arterial blood pressure by circulatory renin-angiotensin	3/30	18/23328	1.55E-06	0.000914	0.000582
	GO:0050872	white fat cell differentiation	3/30	18/23328	1.55E-06	0.000914	0.000582
	GO:0003081	regulation of systemic arterial blood pressure by renin-angiotensin	3/30	33/23328	1.02E-05	0.004019	0.002563
	GO:0001990	regulation of systemic arterial blood pressure by hormone	3/30	47/23328	3.00E-05	0.008851	0.005644
	GO:0003044	regulation of systemic arterial blood pressure mediated by a chemical signal	3/30	55/23328	4.81E-05	0.011377	0.007254
aCD40-aPD-1	GO:0050867	positive regulation of cell activation	115/994	496/23328	2.68E-52	7.92E-49	5.54E-49
	GO:0002696	positive regulation of leukocyte activation	113/994	479/23328	3.30E-52	7.92E-49	5.54E-49
	GO:0001819	positive regulation of cytokine production	109/994	449/23328	1.12E-51	1.80E-48	1.26E-48
	GO:0050900	leukocyte migration	98/994	360/23328	2.44E-51	2.93E-48	2.05E-48
	GO:0007159	leukocyte cell-cell adhesion	96/994	345/23328	3.13E-51	3.01E-48	2.10E-48
CP4	GO:0050727	regulation of inflammatory response	59/511	372/23328	2.41E-33	1.02E-29	7.25E-30
	GO:0050900	leukocyte migration	52/511	360/23328	1.90E-27	4.00E-24	2.85E-24
	GO:0030595	leukocyte chemotaxis	40/511	219/23328	2.88E-25	4.05E-22	2.88E-22
	GO:0060326	cell chemotaxis	44/511	303/23328	1.86E-23	1.96E-20	1.40E-20
	GO:0032103	positive regulation of response to external stimulus	50/511	418/23328	1.17E-22	9.91E-20	7.05E-20
A-aPD-1-T	GO:0050900	leukocyte migration	18/116	360/23328	2.30E-13	3.47E-10	2.59E-10
	GO:0097529	myeloid leukocyte migration	15/116	219/23328	2.86E-13	3.47E-10	2.59E-10
	GO:0097530	granulocyte migration	13/116	155/23328	9.02E-13	7.32E-10	5.46E-10
	GO:1990266	neutrophil migration	12/116	124/23328	1.28E-12	7.76E-10	5.78E-10
	GO:0030593	neutrophil chemotaxis	11/116	99/23328	2.47E-12	1.20E-09	8.97E-10
A-CP4-T	GO:0050900	leukocyte migration	131/2214	360/23328	2.00E-44	1.16E-40	7.21E-41
	GO:0060326	cell chemotaxis	115/2214	303/23328	3.45E-41	9.97E-38	6.21E-38
	GO:0050727	regulation of inflammatory response	122/2214	372/23328	3.48E-36	6.70E-33	4.17E-33
	GO:0097529	myeloid leukocyte migration	90/2214	219/23328	1.16E-35	1.68E-32	1.04E-32
	GO:0030595	leukocyte chemotaxis	89/2214	219/23328	7.95E-35	9.19E-32	5.73E-32
